# PDGFB-targeted functional MRI nanoswitch for activatable T_1_–T_2_ dual-modal ultra-sensitive diagnosis of cancer

**DOI:** 10.1186/s12951-023-01769-7

**Published:** 2023-01-06

**Authors:** Ya’nan Zhang, Lu Liu, Wenling Li, Caiyun Zhang, Tianwei Song, Peng Wang, Daxi Sun, Xiaodan Huang, Xia Qin, Lang Ran, Geng Tian, Junchao Qian, Guilong Zhang

**Affiliations:** 1 School of Medical Imaging, Shandong Technology Innovation Center of Molecular Targeting and Intelligent Diagnosis and Treatment, Binzhou Medical University, Yantai, 264003 People’s Republic of China; 2 School of Pharmacy, Institute of Aging Medicine, Binzhou Medical University, Yantai, 264003 People’s Republic of China; 3 Hefei Cancer Hospital, Anhui Province Key Laboratory of Medical Physics and Technology, Institute of Health and Medical Technology, Hefei Institutes of Physical Science, Chinese Academy of Sciences, Hefei, 230031 People’s Republic of China; 4 Department of Radiation Oncology, School of Medicine, Shandong University, Shandong Cancer Hospital and Institute, Shandong First Medical University and Shandong Academy of Medical Sciences, Jinan, 250117 Shandong China

**Keywords:** Magnetic resonance imaging, TME-activated nanoswitch, Dual-mode contrast agent, Tumor diagnosis, SNR

## Abstract

As one of the most significant imaging modalities currently available, magnetic resonance imaging (MRI) has been extensively utilized for clinically accurate cancer diagnosis. However, low signal-to-noise ratio (SNR) and low specificity for tumors continue to pose significant challenges. Inspired by the distance-dependent magnetic resonance tuning (MRET) phenomenon, the tumor microenvironment (TME)-activated off–on T_1_–T_2_ dual-mode MRI nanoswitch is presented in the current study to realize the sensitive early diagnosis of tumors. The tumor-specific nanoswitch is designed and manufactured on the basis of PDGFB-conjugating ferroferric oxide coated by Mn-doped silica (PDGFB-FMS), which can be degraded under the high-concentration GSH and low pH in TME to activate the T_1_–T_2_ dual-mode MRI signals. The tumor-specific off–on dual-mode MRI nanoswitch can significantly improve the SNR and is used successfully for the accurate diagnosis of early-stage tumors, particularly for orthotopic prostate cancer. In addition, the systemic delivery of the nanoswitch did not cause blood or tissue damage, and it can be excreted out of the body in a timely manner, demonstrating excellent biosafety. Overall, the strategy is a significant step in the direction of designing off–on dual-mode MRI nanoprobes to improve imaging accuracy, which opens up new avenues for the development of new MRI probes.

**Supplementary Information:**

The online version contains supplementary material available at 10.1186/s12951-023-01769-7.

## Introduction

In situ accurate and early diagnosis of cancer is essential for selecting appropriate treatment options and determining prognosis without invasive procedures such as tissue biopsies or surgical excision [1–3]. In recent years, clinical molecular imaging devices, such as X-ray computed tomography (CT), optical imaging, positron emission tomography (PET), and single-photon emission computed tomography (SPECT) techniques, have been utilized extensively to analyze and diagnose tumors in vivo [4, 5]. However, due to the superior soft-tissue contrast, deep tissue penetration, high spatial resolution, and lack of ionizing radiation exposure, etc., magnetic resonance imaging (MRI) can provide anatomical and functional information in diagnosing clinical diseases; consequently, it has demonstrated broader application prospects in the diagnosis of tumors [6–8]. Accordingly, contrast agents, such as T_1_ or T_2_ contrast nanomaterials, have been developed for MRI over the past few decades [9–11]. However, single-mode MR contrast agents (CAs) have inherent drawbacks, such as a short blood circulation time, magnetic susceptibility artifacts, and a clinical accuracy of tumor diagnosis that is not always met [12–14]. Therefore, it is of the utmost importance to design versatile contrast nanoprobes capable of overcoming limitations to achieve accurate diagnostic results.

The concurrent use of T_1_–T_2_ dual-mode imaging modalities has been reported for the cross-validation of acquired image data [15, 16]. Accordingly, this strategy combines the advantages of two single-mode imaging techniques to provide extremely precise image information. Based on the distance-dependent magnetic resonance tuning (MRET) phenomenon, however, when the enhancer is located close to the quencher, the electron spin fluctuation rate of the enhancer decreases, preventing it from accelerating and relaxing water protons, thereby resulting in a quenched longitudinal relaxivity (r_1_) [17–19]. Additionally, Due to direct contact between the two agents, the magnetic field derived from the superparamagnetic T_2_ CA disrupts the relaxation process of the paramagnetic T_1_ CA, resulting in the attenuation of the T_1_ signal and a significant decline in the T_1_–T_2_ contrast enhancement [20]. Conversely, “always on” MRI contrast agents frequently disregard the specific interaction with tumor tissues exhibiting weak acidity and high GSH concentration, resulting in a low signal-to-noise ratio (SNR) [21–23]. Thus, the development of a smart stimuli-activated MRI off–on nanoswitch [24, 25] to meet the practical requirements for early, tumor-specific diagnosis, with high SNR and off–on properties, presents a significant challenge.

**Scheme 1.**
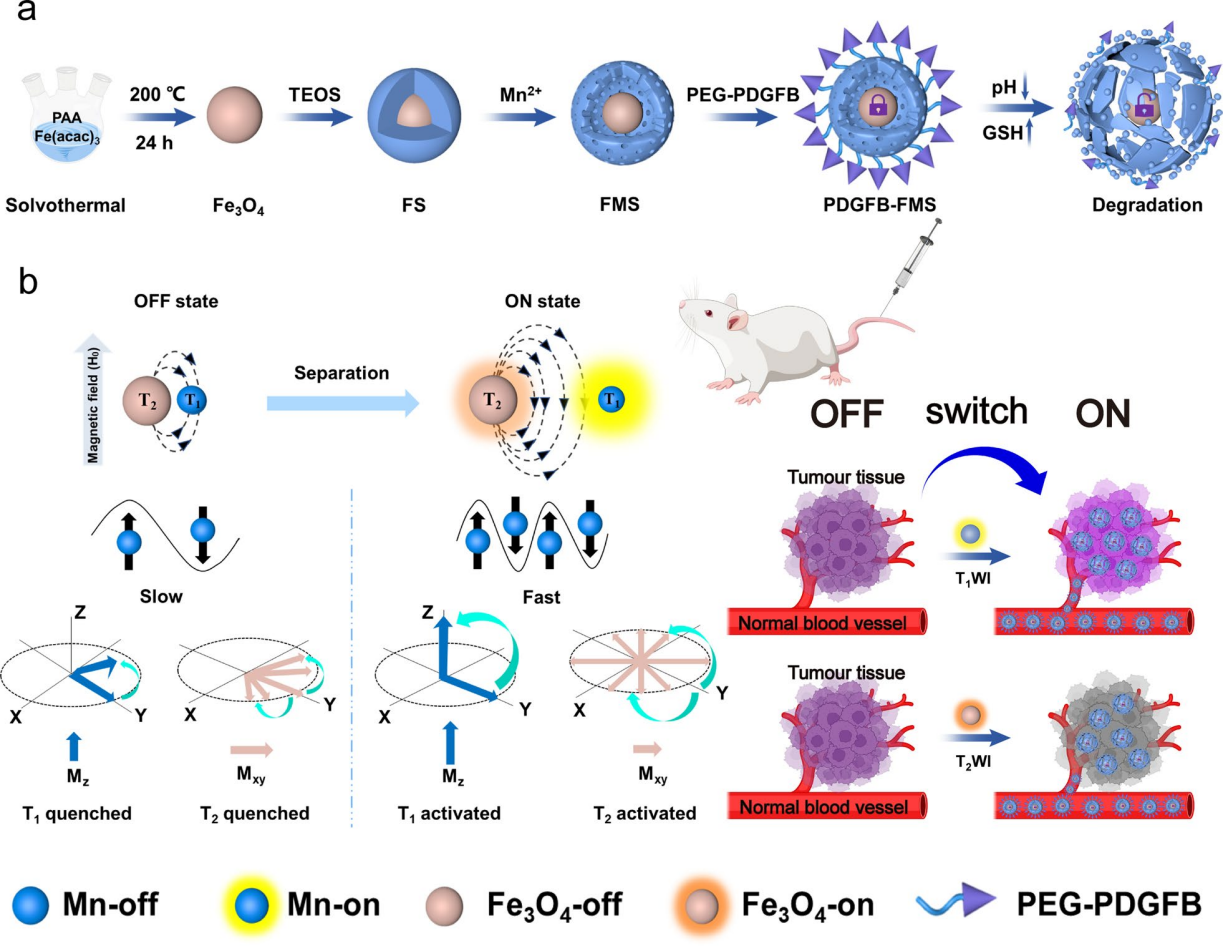
**a** Schematic illustration of synthetic procedures of PDGFB-FMS and **b** the mechanism of PDGFB-FMS as the intelligent bimodal MRI contrast agents

In this study, inspired by the MRET principle, a novel tumor microenvironment (TME)-activated T_1_- and T_2_-dual-mode MRI nanoswitch was designed using platelet-derived growth factor (PDGFB)-conjugated ferroferric oxide coated by Mn-doped silica (FMS) yolk-shell nanostructures (PDGFB-FMS) (Scheme 1), which display a distinct "off–on" T_1_–T_2_ dual-modal synergistic imaging with an ultrafast response, high sensitivity and specificity toward TME. Under normal tissue conditions, this nanostructure for dual-mode imaging is so stable that the T_1_ and T_2_ MR signals initially display an "off" state. However, when the tumor is targeted, the weakly acidic and high GSH of TME disrupts the PDGFB-FMS, causing its nanostructure to collapse, resulting in the rapid release of Mn^2+^ ions, which separates from the Fe_3_O_4_ magnetic core and activates the dual-mode of the T_1_ and T_2_ MRI signals. In addition, the conjugation of PDGFB cycle peptides makes the nanoswitch recognize the tumor site specifically [26–32], allowing the nanoswitch to be effectively internalized by tumor cells and accumulate preferentially at tumor sites. This smart activatable dual-mode MRI nanoswitch not only responds specifically to TME but also exhibits no signal (or a very weak signal) in normal tissues, which can reduce background noise and improve SNR. We, therefore, anticipate that the dual-mode nanoprobes will play a major role in the development of a variety of diagnostic nanoplatforms.

## Results and discussion

### Synthesis of FMS yolk-shell nanoswitch

**Fig. 1:**
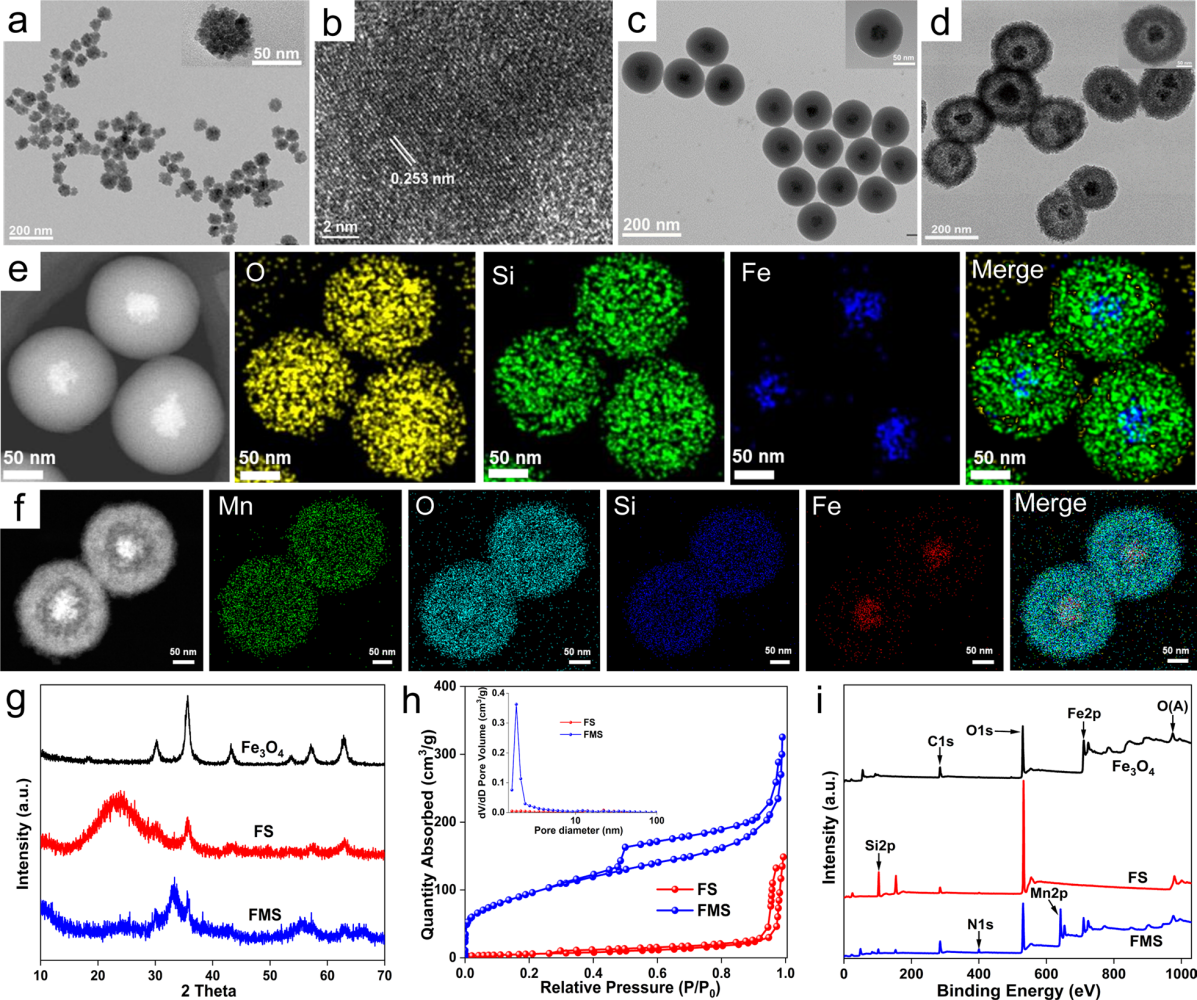
**a** TEM image and **b** HRTEM image of Fe_3_O_4_. TEM images of **c** FS and **d** FMS. HAADF-STEM and elemental distribution images of **e** FS and **f** FMS. **g** XRD patterns of Fe_3_O_4_, FS, and FMS. **h** N_2_ adsorption/desorption isotherms of FS and FMS (inset: pore size distribution), **i** XPS full spectra of Fe_3_O_4_, FS, and FMS

The preparation of FMS consisted of three steps, as illustrated in Scheme 1a. We first synthesized Fe_3_O_4_ nanoclusters via the solvent thermal decomposition method using ferric acetylacetonate as the iron source and sodium acrylate as the template. As depicted in Fig. 1a, the Fe_3_O_4_ nanoclusters were monodispersed and uniformly spherical with a size of approximately 60 nm. Moreover, the hydrodynamic diameter of the Fe_3_O_4_ nanocluster was approximately 60 ± 19.7 nm, which is comparable to the size obtained from transmission electron microscopy (TEM) studies, indicating that the Fe_3_O_4_ nanocluster had an excellent colloidal performance. In addition, the high-resolution TEM (HRTEM) image reveals that the Fe_3_O_4_ nanoclusters were highly crystalline, with a lattice fringe distance of ~ 0.253 nm, which corresponds to the (311) atomic plane of magnetite (Fig. 1b). Subsequently, the well-dispersed spherical Fe_3_O_4_@SiO_2_ (FS) core–shell nanoparticles with a shell thickness of ~ 35 ± 16.0 nm were formed by using TEOS as the silica source to coat the Fe_3_O_4_ under heating and alkaline conditions (Fig. 1c). Finally, a specific “ammonia-assisted Mn^2+^ etching” strategy was used to etch the outer silica shell to form Mn-doped silica-coated ferroferric oxide nanoparticles (FMS) via the Ostwald ripening mechanism [33]. Compared to untreated FS nanoparticles, the representative TEM image revealed well-defined yolk-shell nanostructures with a larger size (~ 220 ± 100.8 nm) (Fig. 1d). In addition, the FMS exhibited a porous structure that was loose and rough, providing an abundance of active sites for proton exchange. Furthermore, the increasing hydrodynamic size of nanoparticles from FS to FMS also confirmed the TEM observations (Additional file 1: Fig. S1). The high-angle annular dark field scanning TEM (HAADF-STEM) image of FS revealed the composition and nanostructure of the core–shell, but FMS further illustated the successful doping of Mn ions and nanostructure changes from core–shell to yolk-shell (Fig. 1e, f). Moreover, the energy-dispersive X-ray (EDX) spectra showed that all expected elements (Fe, Si, Mn, and O) were detected and their relative positions in yolk-shell nanostructures are well matched (Additional file 1: Fig. S2g–i).

In addition, X-ray diffraction (XRD) spectra of Fe_3_O_4_ revealed the characteristic spinel structure of magnetite (Fig. 1g) [34]. Upon growth of the SiO_2_ shell layer, a characteristic broad peak of amorphous silica is produced, whereas it nearly disappears after Mn^2+^ ions etch the FMS. This result indicates that the silica shell is successfully coated on the surface of Fe_3_O_4_ nanoparticles, and it is effectively etched by Mn^2+^ in the subsequent process. After Mn^2+^ etching, significant increases in specific surface area (342.92 m^2^/g) and porosity are observed for FMS nanostructures with a pore size distribution dominated by 2.0 nm (Fig. 1h). As is well known, the weight loss of the thermogravimetric (TG) curves below 200 °C is attributable to the loss of water molecules, including physically adsorbed and bound water. Accordingly, the TG analysis suggests that FMS adsorbs significantly more water (7.98%) than FS (6.58%) and Fe_3_O_4_ (2.35%) (Additional file 1: Fig. S2a). These findings indicate that FMS has a strong affinity for water molecules and facilitates the interaction with the proton, which can improve the relaxation properties.

In the Fourier transform infrared (FT-IR) spectrum of Fe_3_O_4_, the peak of the Fe–O bond appears at 570 cm^−1^ [34], while the typical peaks of the –C=O and C–H bonds appear at 1635 cm^−1^ and 2920 cm^–1^, respectively (Additional file 1: Fig. S2e). In addition, the FT-IR spectrum of FS exhibits a distinct Si–O peak at 1100 cm^−1^ [35], indicating that silica has been successfully coated on the surface of Fe_3_O_4_. Moreover, the Si–O peak of FMS shifts significantly from 1100 to 1000 cm^−1^ as a result of the transformation of the Si–O-Si bond to the Mn–O-Si bond, indicating the successful etching of the silica layer. In the PDGFB-FMS FT-IR curve, the new peak at 1450 cm^−1^ indicates that PDGFB is successfully anchored to the surface of FMS. The formation of PDGFB-FMS nanoswitch is further supported by ζ potential changes of particles (Additional file 1: Fig. S2f). The outer SiO_2_ layer of the FS exhibits a negative ζ potential of approximately − 46.3 mV. In contrast, the ζ potential of FMS decreased to − 15.5 mV, confirming the presence of Mn^2+^ ions. Furthermore, the ζ potential of PDGFB-FMS was found to be − 18 mV, indicating that the conjugation of PDGFB was successful. The presence of Fe, Si, Mn, C, and O was further confirmed from the full XPS spectrum of FMS (Fig. 1i). Due to the dense, thick SiO_2_ shell that obscured the Fe signal in the Fe2p high-resolution spectrum of FS, Fe2p peaks at 711.8 and 724.3 eV vanished. Notably, the Fe peaks of the FMS yolk-shell nanostructure recovered, and strong Mn peaks emerged (Fig. 1i and Additional file 1: Fig. S2b, d), indicating the formation of a loose Mn-doped silica shell layer. Remarkably, the Si2p peak for FS shifted significantly from 103.3 to 102.4 eV (Additional file 1: Fig. S2c), further confirming the transformation of Si from a dense silica layer to a loose structure. The hydrodynamic size of PDGFB-FMS had no significant variation in blood, phosphate-buffered saline (PBS), and 10% fetal bovine serum (FBS) within 3 days, demonstating excellent stability (Additional file 1: Fig. S3).

### TME-responsive “off–on” T_1_–T_2_ imaging of FMS nanoswitch

**Fig. 2:**
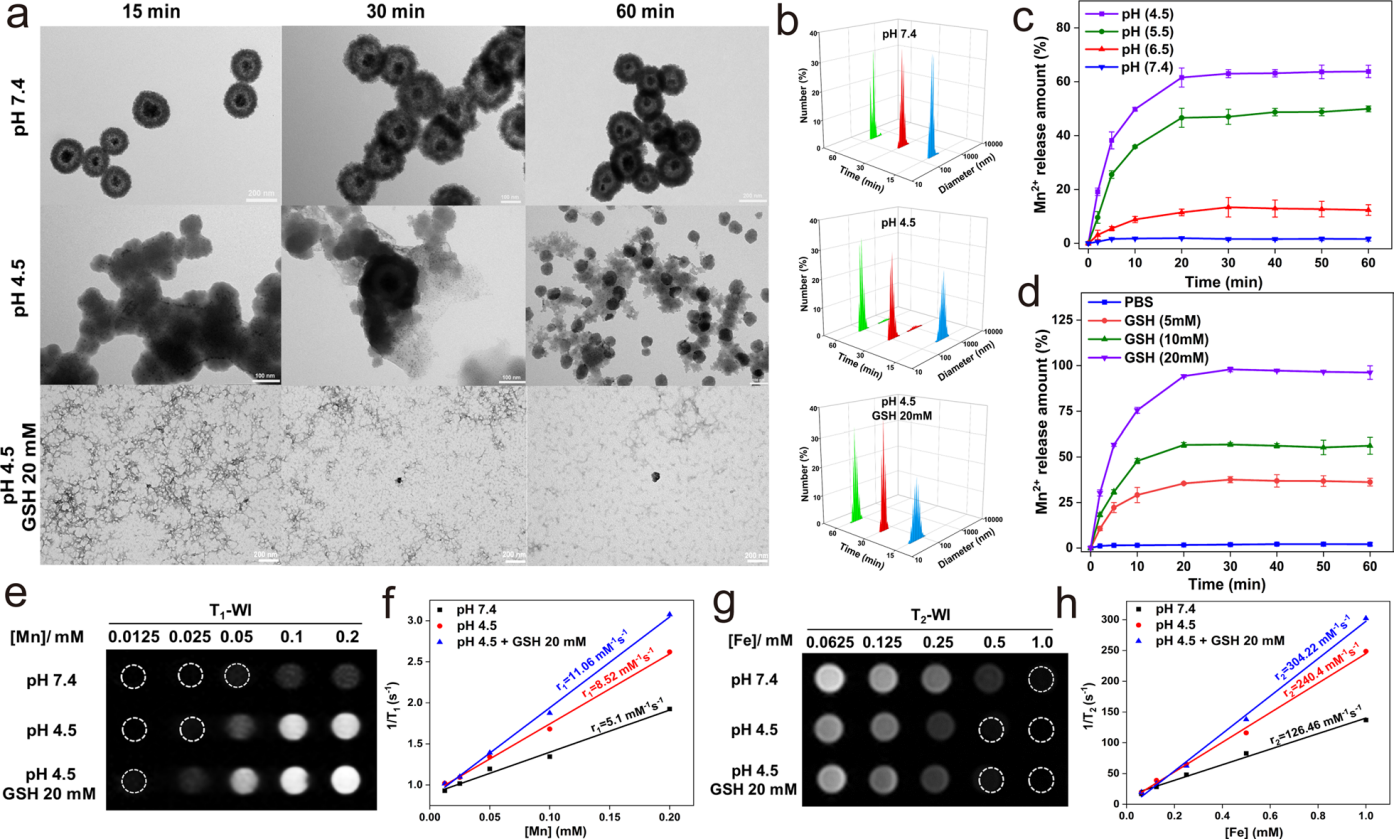
**a** TEM images and **b** corresponding hydrodynamic size changes (blue, 15 min; red, 30 min; green, 60 min) of FMS after treated with different pHs and GSH conditions. **c**, **d** Cumulative Mn^2+^ release curves of FMS at different **c** pH and **d** GSH concentrations. **e** T_1_WI and **f** corresponding r_1_ values of the FMS phantoms after different treatments under 3.0 T. **g** T_2_WI and **h** corresponding r_2_ values of the FMS phantoms after different treatments under 3.0 T

We also analyzed the degradation of FMS under GSH and weak acid conditions. In the presence of weak acidity and GSH, Fig. 2a, b illustrates the significant effects of FMS on the hydrodynamic size distribution and morphology of corresponding particles. Most FMS particles disassemble from regular spherical structures into scattered dots over time. In contrast, no discernible change is observed for FMS in the absence of GSH and under neutral conditions. In addition, the degree of dissociation of FMS increased significantly with increasing GSH concentration and decreasing pH, demonstrating excellent pH- and GSH-responsive degradability (Additional file 1: Fig. S4). To gain more insight into the evolution process, the release of Mn^2+^ ions from FMS with the change of GSH concentration and pH value was investigated. Accordingly, the results confirm that the behavior of Mn^2+^ ion release from FMS is pH- and GSH-dependent (Fig. 2c, d). These results indicate that FMS can be responsively dissociated in TME with an abundance of GSH and an acidic environment by breaking the Mn–O bond and the subsequent collapse of the FMS backbone within one hour (Fig. 2c, d). Based on the excellent degradation of FMS triggered by TME, we further investigated the off–on switchable properties of the dual-mode T_1_–T_2_ signal in FMS. Under physiological conditions (in pH 7.4 PBS), both T_1_- and T_2_-weighted imaging (T_1_WI and T_2_WI, respectively) exhibited an apparent quenching phenomenon (Fig. 2e, g). In addition, the corresponding T_1_ relaxivity (r_1_) and T_2_ relaxivity (r_2_) were measured to be 5.1 and 126.46 mM^−1^ s^−1^, respectively (Fig. 2f, h). When exposed to GSH (20 mM) and pH 4.5, the FMS nanostructure decomposed rapidly into Fe_3_O_4_ and Mn^2+^ ions, resulting in a rapid recovery of T_1_ and T_2_ signals as the distance between the Mn^2+^ ions and Fe_3_O_4_ magnetic core increased (Fig. 2e, g). After treatment with PBS containing 20 mM GSH and pH 4.5, the calculated r_1_ and r_2_ values were found to be 11.06 and 304.22 mM^−1^ s^−1^, respectively (Fig. 2f, h). In addition, as depicted in Additional file 1: Figs. S5, S6, the “off–on” characteristics of dual-mode T_1_–T_2_ nanoprobes were highly dependent on GSH concentrations and pH values. These results, therefore, support our hypothesis that low pH and high GSH concentrations triggered the release of Mn^2+^, which reduces the mutual magnetic resonance interference between Mn^2+^ and Fe_3_O_4_ and activates the T_1_–T_2_ MRI signals (Additional file 1: Tables S1 and S2).

### Quenching mechanism of T_1_ and T_2_ imaging

**Fig. 3:**
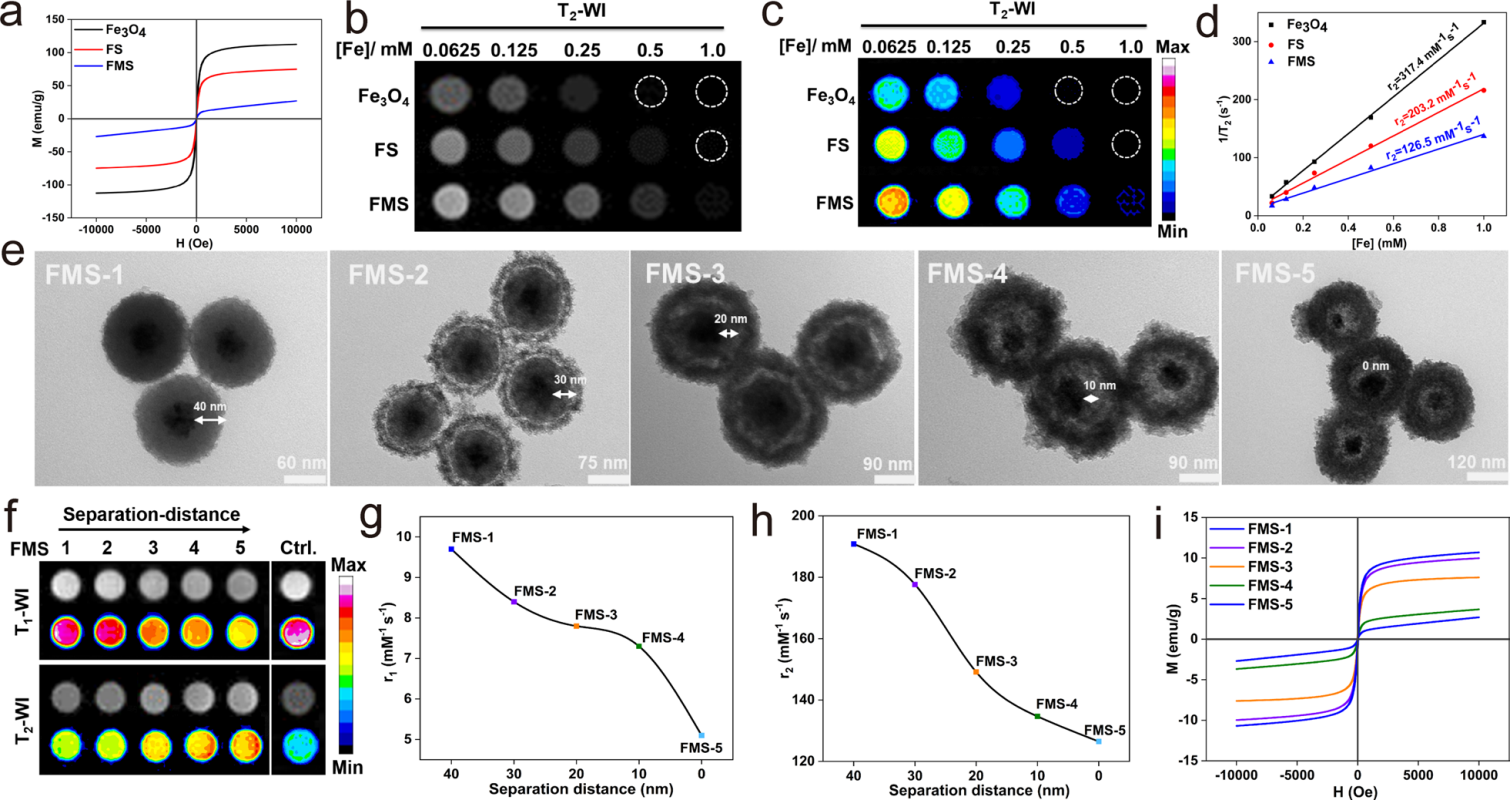
**a** M-H hysteresis loops of Fe_3_O_4_, FS, and FMS at 300 K. **b** Grayscale T_2_WI, and **c** pseudocolor T_2_WI, and **d** corresponding r_2_ values of Fe_3_O_4_, FS, and FMS. **e** TEM images, **f** T_1_WI and T_2_WI, **g** r_1_ values, **h** r_2_ values, and **i** M-H hysteresis loops at 300 K of FMS-1 to FMS-5

The T_1_–T_2_ dual-quenching mechanism of FMS was also investigated methodically. Accordingly, Mn^2+^ ions with a single T_1_ contrast and Fe_3_O_4_ with a single T_2_ contrast were used as controls (Additional file 1: Fig. S7a and Fig. 3). For the T_1_ quenching effect, we proposed that the strong local magnetic field originating from the large magnetic moment of the Fe_3_O_4_ magnetic core would interfere with the spin–lattice relaxation process of Mn-doped silica shell with much weaker paramagnetism surrounding it, thereby quenching the T_1_ signal of paramagnetic Mn^2+^. Accordingly, the M-H curves of FMS exhibited a lower saturation magnetization (25 emu/g) at 300 K in comparison to Fe_3_O_4_ (112.4 emu/g). In addition, the magnetic field generated by the superparamagnetic Fe_3_O_4_ core significantly disrupts the relaxation process of the paramagnetic Mn-doped silica shell, resulting in the quenching of the T_1_ signal during the etching procedure (Fig. 3a). Meanwhile, the grayscale and corresponding pseudo-color T_2_WI of Fe_3_O_4_, FS, and FMS exhibited significant T_2_ quenching effects (Fig. 3b, c), and their T_2_ relaxivities (r_2_) were 317.4 mM^−1^ s^−1^, 203.2 mM^−1^ s^−1^, and 126.5 mM^−1^ s^−1^, respectively (Fig. 3d). Therefore, T_1_ and T_2_ MRI signals can be interfered with when Fe_3_O_4_ and Mn-doped silica are in direct contact. To further validate the MRET hypothesis, we synthesized a series of FMS (FMS-1, FMS-2, FMS-3, FMS-4, FMS-5) with different separation distances between the paramagnetic Mn^2+^ and the Fe_3_O_4_ magnetic core by varying the thickness of the silica separation layer from 40 ± 2.6 to 0 ± 1.2 nm (Fig. 3e). Notably, the hydrodynamic size of FMS increased gradually as the etching process progressed (Additional file 1: Fig. S7b), while simultaneously increasing the specific surface area and porosity (Additional file 1: Fig. S8), facilitating the energy exchange with water molecules. The nanoswitch showed obvious quenching in T_1_ and T_2_ signals with the Mn^2+^ doping ratio from 33 to 88% (Additional file 1: Fig. S9). This was because that high ratio of Mn-doped silica significantly reduced the distance between Fe_3_O_4_ core and Mn^2+^, resulting in the increase of quenching effect in T_1_ and T_2_ imaging. As shown in Fig. 3f, the T_1_ MRI signal of the Mn-doped silica shell in FMS decreases as the Mn approaches the Fe_3_O_4_ core. Furthermore, as the separation distance decreases from 40 to 30, 20, 10, and 0 nm, the r_1_ values of the Mn-doped silica shell decrease from 9.7 to 8.4, 7.8, 7.3, and 5.1 mM^−1^ s^−1^ (Fig. 3g and Additional file 1: Fig. S7c), which is lower than that of free Mn^2+^ (r_1_ = 15.9 mM^−1^ s^−1^) (Additional file 1: Fig. S7a). Concurrently, a similar T_2_ MRI signal quenching phenomenon was observed in T_2_WI and corresponding pseudo-color MR images (Fig. 3f). As the etching depth increases, the r_2_ value of Fe_3_O_4_ decreases from 190.85 to 177.66, 149.15, 134.67, and 126.5 mM^−1^ s^−1^ (Fig. 3h and Additional file 1: Fig. S7d). In addition, the saturated magnetization of FMS decreased gradually with increasing Mn content, which could be attributed to the disruption of Fe_3_O_4_ magnetic moments induced by paramagnetic Mn^2+^ (Fig. 3i). Thus, the aforementioned results demonstrate that the T_1_ and T_2_ dual-quenching effect of FMS is inversely proportional to the separation distance, which can be precisely controlled by the Mn etching process.

### Evaluation of internalization and cell MRI of PDGFB-FMS

**Fig. 4:**
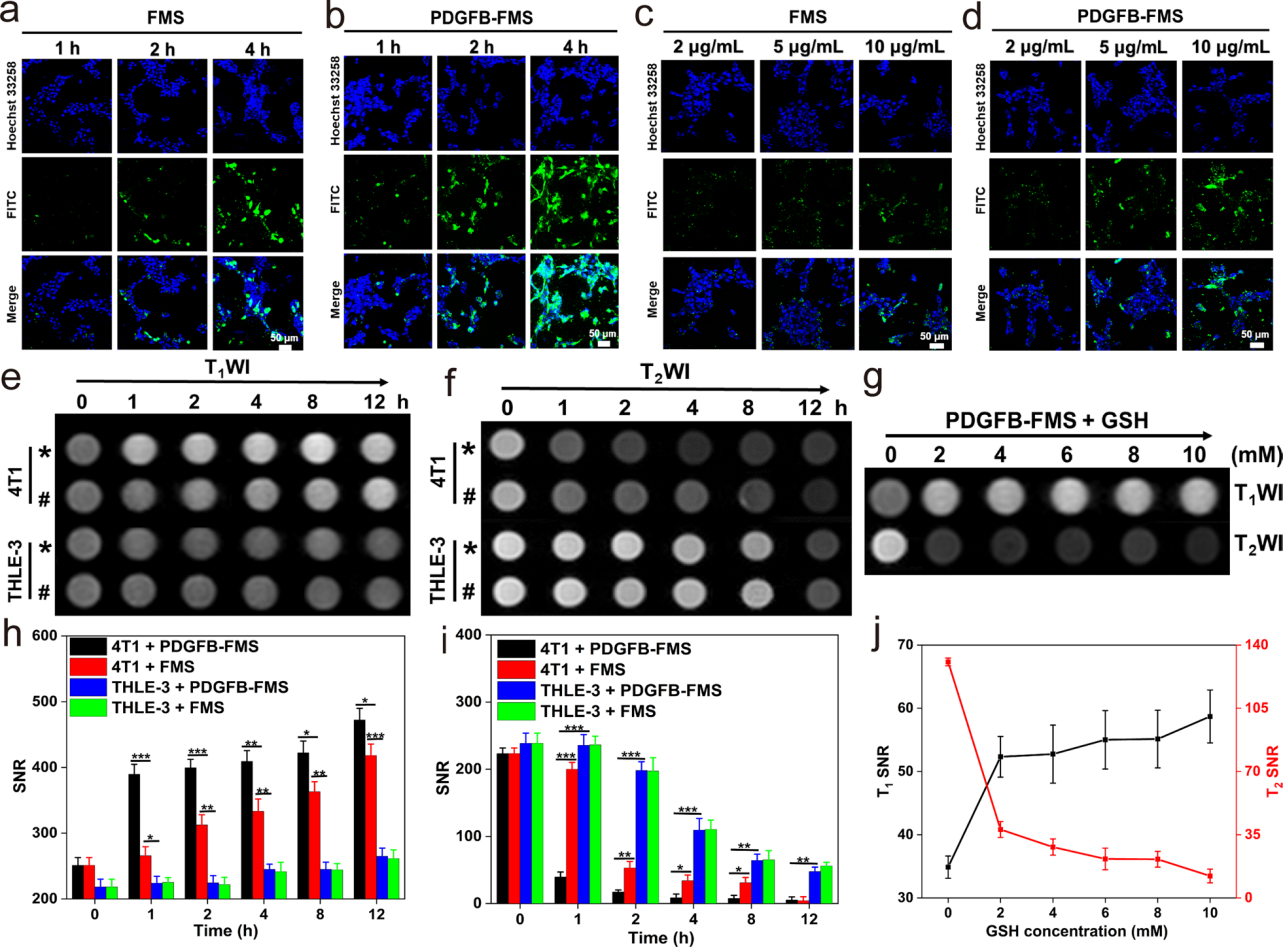
**a**, **b** CLSM observation: the internalization process of 4T1 cells treated with FMS and PDGFB-FMS at a certain concentration of 10 μg/mL for 1, 2, and 4 h. **c**, **d** CLSM observation: the internalization process of 4T1 cells treated with different concentrations of FMS and PDGFB-FMS for 4 h. **e** T_1_WI and **f** T_2_WI of the cells treated with different samples (* and # represent PDGFB-FMS and FMS, respectively) at different time points, and **h**, **i** the corresponding T_1_ and T_2_ MRI SNR analysis. **g** T_1_WI and T_2_WI of 4T1 cells treated with PDGFB-FMS at the presence of different concentrations of GSH, and **j** the corresponding T_1_ and T_2_ MRI SNR analysis

The hypoxic TME stimulates the overexpression of PDGF-β receptors in breast cancer cells and prostate cancer cells [36, 37]. To obtain tumor-specific delivery, the PDGFB targeting ligand is, therefore, conjugated to FMS to create a PDGFB-FMS nanoswitch that targets tumors. The internalization efficacy of a nanoprobe is essential for evaluating its targeting capability and contrast performance. Using confocal laser scanning microscopy (CLSM) and inductively coupled plasma mass spectrometry (ICP-MS) analysis, the uptake of FMS and PDGFB-FMS was, therefore, investigated in 4T1 cells and PC-3 cells based on this information (Fig. 4a–d, and Additional file 1: Figs. S10, S11). The results of CLSM indicated that the internalization of FMS and PDGFB-FMS is concentration- and time-dependent. In addition, PDGFB-FMS treated cells exhibited a stronger green fluorescence than FMS-treated cells, confirming their superior targeting ability to breast cancer and prostate cancer. We also calculated the colocalization and particle uptake ratios corresponding to the CLSM images. We found that the differences in cell internalization for FMS and PDGFB-FMS treatments are statistically significant (Additional file 1: Fig. S12). In addition, Fe content was used as an indicator for ICP-MS analysis to determine the internalization efficacy of PDGFB-FMS. Results indicate that Fe content in 4T1 cells increased gradually with increasing time and concentration, and cells treated with PDGFB-FMS accumulated more Fe than those treated with FMS. These results are consistent with the CLSM observation, further validating the performance of PDGFB-FMS in targeting tumors.

The imaging performance of PDGFB-FMS was further investigated at the cellular level, in light of the aforementioned encouraging findings. The T_1_ and T_2_-weighted signals of 4T1 cells treated with FMS and PDGFB-FMS were found to be significantly activated (Fig. 4e, f, h, i) and demonstrate a time-activation relationship. Notably, the T_1_ and T_2_ signal almost remained unchanged in THLE-3 cells incubated with FMS and PDGFB-FMS, which can be attributed to the lower cellular GSH level and increased internalization efficacy. To determine whether the activation of PDGFB-FMS correlates positively with intracellular GSH levels, 4T1 cells were pretreated with various concentrations of GSH and then incubated continuously with PDGFB-FMS. The results revealed that both the T_1_ and T_2_ signals of cells were significantly improved (Fig. 4g, j). Moreover, the corresponding relaxation rate change ΔR_1_ (R_1_ = 1/T_1_) and ΔR_2_ (R_2_ = 1/T_2_) increased gradually after GSH pretreatment (Additional file 1: Table S3), suggesting that the intracellular high level of GSH can be used as a stimulus to activate the dual-mode MRI contrast enhancement.

### In vivo MRI studies of PDGFB-FMS

**Fig. 5:**
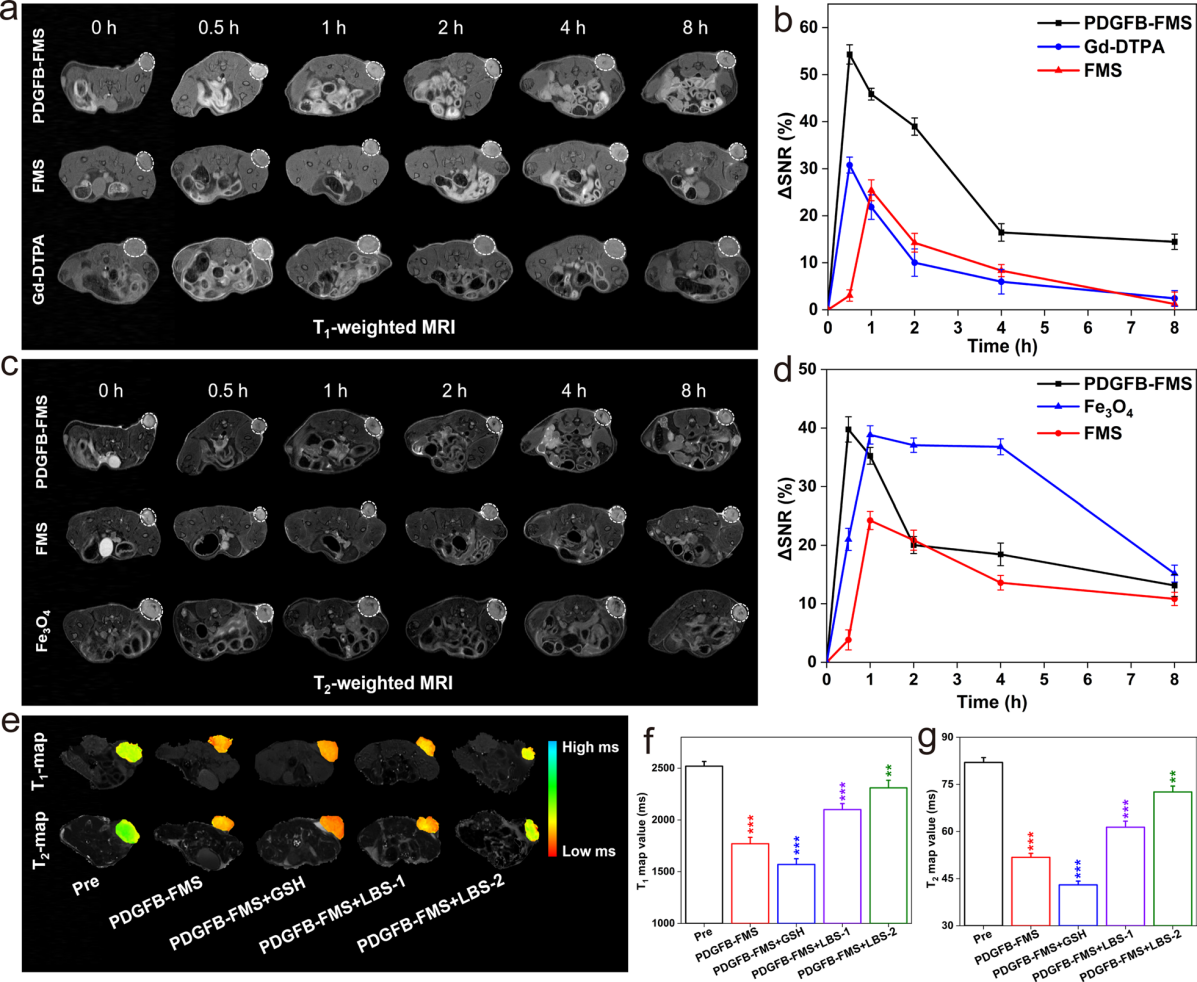
**a**, **c** Representative T_1_WI and T_2_WI of 4T1 tumor-bearing mouse at axial planes after intravenous injection of different samples and **b**, **d** ∆SNR analysis corresponding to **a**, **c**, respectively. Tumor region are marked by white dotted lines. **e** color-coded T_1_ and T_2_ map images, **f** corresponding T_1_ map value, and **g** T_2_ map values of 4T1 tumor-bearing mice treated with PDGFB-FMS at the presence of GSH or GSH inhibitor (LBS)

A series of in vivo MRI experiments were also designed and conducted using a mouse model bearing the 4T1 tumor to validate the activable MRI diagnosis for the tumor. FDA-approved “Magnevist” (Gd-DTPA) and “Feridex” (Fe_3_O_4_) were selected as controls for T_1_WI and T_2_WI, respectively. Accordingly, the 4T1 tumor-bearing mice were intravenously administrated using Gd-DTPA, Fe_3_O_4_, FMS, and PDGFB-FMS at the dosage of 5 mg/kg, respectively, following which, they were scanned using a 7.0 T MRI scanner, and the T_1_WI and T_2_WI of the tumor in the axial plane were obtained at various time points. As shown in Fig. 5a, the T_1_WI of tumors treated with PDGFB-FMS brightens significantly at 0.5 h post-injection (p.i.) and then darkens over time. In addition, the tumor site of the PDGFB-FMS group in T_1_WI was observed to be the clearest and brightest compared to those of FMS and Gd-DTPA. Similarly to T_1_WI, the T_2_WI of the tumor treated with PDGFB-FMS was observed to be darkest at p.i. 0.5 h (Fig. 5c), following the same pattern as T_1_WI. On the contrary, the contrast enhancement of FDA-approved Gd-DTPA and Fe_3_O_4_ for T_1_WI and T_2_WI, respectively, was found to be weak. In addition, the quantitative analysis of tumor regions is performed by measuring the change in SNR (ΔSNR) before and after administration. As shown in Fig. 5b, the maximum ΔSNR of the tumor regions in the PDGFB-FMS group was greater than that of the FMS and Gd-DTPA groups, demonstrating the most pronounced contrast enhancement. In addition, T_2_WI of mice treated with FMS exhibited a low ΔSNR, whereas, PDGFB-FMS exhibited a comparable ΔSNR (Fig. 5d), which may be a result of efficient targeting. In addition, the respective T_1_ and T_2_ map images confirmed the aforementioned findings. As shown in Additional file 1: Fig. S13, there was a 30.7% decrease in the T_1_ map value in the tumor region treated with PDGFB-FMS at p.i. 0.5 h, whereas the Gd-DTPA and FMS groups only led to a 16.5% and 16.6% decrease, respectively. Similarly, a significant decrease in the T_2_ map value is observed at p.i. 0.5 h, and it follows the same pattern as the T_1_ map value. The aforementioned results demonstrate that the PDGFB-FMS dual-activated nanoswitch has the desired dual-modal imaging capability for accurate tumor diagnosis.

To further illustrate GSH tuning nanoswitch, we examined the T_1_ and T_2_ tumor map images following PDGFB-FMS treatment in the absence and presence of GSH and GSH inhibitor (LBS). Briefly, mice carrying 4T1 tumors were intratumorally pretreated with 10 mM GSH, 2.5 mM LBS (LBS-1), or 5 mM LBS (LBS-2), respectively. The PDGFB-FMS was then administered intravenously to these 4T1 tumor-bearing mice. As anticipated, the GSH-pretreated tumor exhibited lower T_1_ and T_2_ map values at the same PDGFB-FMS injection as compared to the non-GSH-pretreated tumor. In contrast, T_1_ and T_2_ map value attenuation of the tumor after LBS pretreatment was significantly inhibited, and their inhibition effect was found to be concentration-dependent (Fig. 5e–g). This phenomenon indicates that the decreased intratumoral GSH concentration caused by LBS prevented the activation of GSH-activated T_1_–T_2_ signals. The findings, therefore, clearly demonstrate the clear positive correlation between PDGFB-FMS activation and intracellular GSH concentrations.

**Fig. 6:**
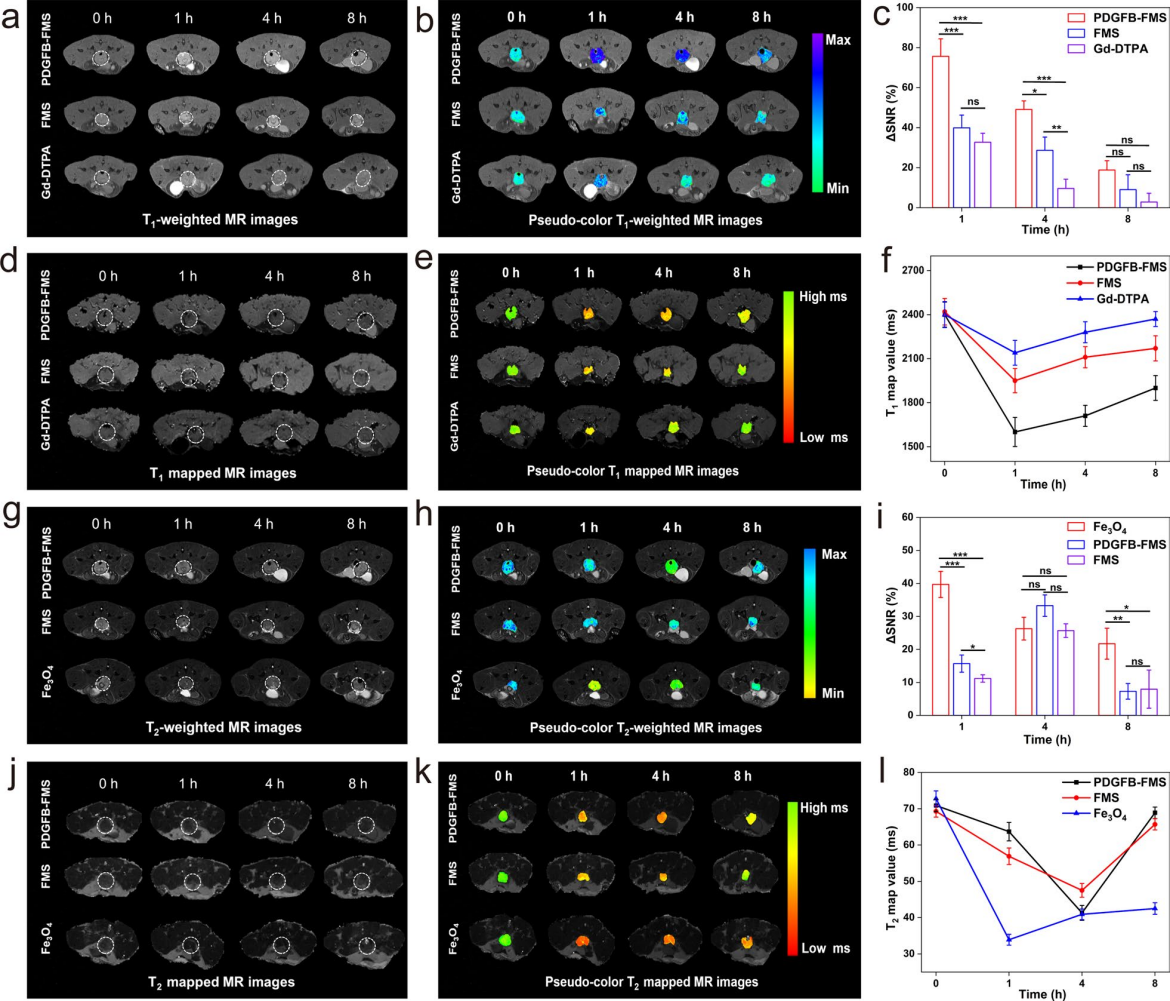
MRI visualization of early orthotopic prostate tumor-bearing mice treated with different samples. **a** Grayscale and **b** pseudocolor T_1_WI. **c** ΔSNR corresponding to **a**. **d** Grayscale and **e** color-coded T_1_ map images. **f** T_1_ map values analysis corresponding to **d**. **g** Grayscale and **h** pseudocolor T_2_WI. **i** ΔSNR corresponding to **g**. **j** Grayscale and **k** color-coded T_2_ map images. **l** T_2_ map values analysis corresponding to **j**. Tumor regions are indicated by white dotted lines

The imaging potential of the PDGFB-FMS nanoswitch for in-situ carcinoma was also investigated (Fig. 6). This study establishes the transgenic adenocarcinoma of mouse prostatic (TRAMP) model to simulate the progression of orthotopic prostate cancer [38, 39]. We first assessed the cytotoxicity of PDGFB-FMS nanoswitch via cell counting kit-8 (CCK-8) assays before in vivo MRI studies. No apparent cytotoxicity against PC-3 cells was observed for PDGFB-FMS nanoswitch, indicating that PDGFB-FMS nanoswitch has good biocompatibility (Additional file 1: Fig. S14). Then, transgenic adenocarcinoma mouse prostate (TRAMP) mice were administered Gd-DTPA, Fe_3_O_4_, FMS, and PDGFB-FMS intravenously, respectively. Correspondingly, the T_1_ and T_2_ MR contrasts of the prostate tumor site were significantly enhanced 1 and 4 h post-injection of PDGFB-FMS, respectively. However, the modest dynamic contrast enhancement of T_1_ and T_2_ in the Gd-DTPA, Fe_3_O_4_, and FMS groups was visible 8 h after injection (Fig. 6a, b, g, h). The ΔSNR was then used to quantify the MR signals of tumors at different time points (Fig. 6c, i). Accordingly, for T_1_-weighted MRI, the maximum ΔSNR of tumors treated with PDGFB-FMS reached up to 75.6 ± 8.8%, which was significantly greater than that of tumors treated with FMS (39.9 ± 6.3%) and Gd-DTPA (32.7 ± 4.5%) (Fig. 6c). Using T_2_-weighted MRI, the maximal ΔSNR of tumors treated with PDGFB-FMS, FMS, and Fe_3_O_4_ is 33.3%, 25.7%, and 39.7%, respectively (Fig. 6i). These results suggest that PDGFB-FMS targets tumor sites and that T_1_ and T_2_ imaging are effectively illuminated by TME. Furthermore, we collected T_1_ and T_2_ map images to verify this conclusion (Fig. 6d, e, j, and k). At the tumor treated with PDGFB-FMS, approximately 33.3% of reduction in T_1_ map value was detected, whereas FM and Gd-DTPA only led to 19.4% and 10.8% reduction in T_1_ map value, respectively (Fig. 6f). Moreover, PDGFB-FMS exhibited a significant decrease in T_2_ map value (41.7% at p.i. 4 h), whereas T_2_ map value changes for FMS and Fe_3_O_4_ were 31.9% and 53.0%, respectively (Fig. 6l). Considering the significantly higher T_1_ and T_2_ ΔSNR and the sensitive “off–on” dual-mode MRI signals, the PDGFB-FMS nanoswitch demonstrates its superiority in the area of early-stage cancer detection.

### In vivo biosafety assessment of PDGFB-FMS nanoswitch

**Fig. 7:**
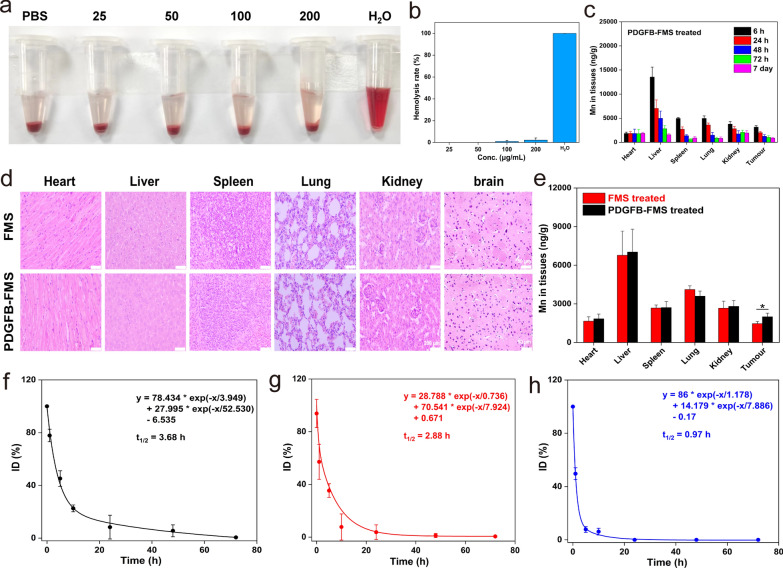
**a** Hemolysis photos and **b** hemolysis rate of PDGFB-FMS. **c** Bio-distribution of Mn element after i.v. injection of PDGFB-FMS. **d** H&E staining of major organs in the mice treated with FMS and PDGFB-FMS for 7 days. The scale is 50 μm. **e** Bio-distributions of Mn element in major organs after treated with PDGFB-FMS and FMS for 24 h. Pharmacokinetic curves of **f** PDGFB-FMS, **g** FMS, and **h** Gd-DTPA in vivo

As is well-known, the biosafety of a nanoagent is a crucial factor to consider when assessing its clinical application potential [40, 41]. Based on this, we first evaluate the biocompatibility of PDGFB-FMS with blood using a hemolysis assay and a routine blood examination. As shown in Fig. 7a and b, the hemolysis rate of PDGFB-FMS is extremely low and negligible, indicating that PDGFB-FMS cannot damage red cells. In addition, major blood routine indicators such as WBC, RBC, PLT, HGB, and HCT (Additional file 1: Fig. S15) at p.i. PDGFB-FMS did not demonstrate any significant changes. These results demonstrate the biocompatibility of PDGFB-FMS with blood. Subsequently, we also investigated cytoxicity of PDGFB-FMS on normal cells (THLE-3, 293T, PC-12, and SH-SY5Y cells). As shown in Additional file 1: Fig. S16a–c, the PDGFB-FMS is biocompatible with THLE-3, 293T, PC-12, and SH-SY5Y cells over a wide range of concentrations, exhibiting no toxicity. We also explored tissue toxicity of PDGFB-FMS using hematoxylin and eosin (H&E) analysis. After 7 days of PDGFB-FMS treatment, no obvious abnormalities in the vital organs were observed, confirming the excellent biosafety of the tissue (Fig. 7d). In addition, the biodistribution analysis revealed that the reticuloendothelial system was responsible for the majority of PDGFB-FMS accumulation in the liver (Fig. 7c). Notably, PDGFB-FMS levels in the body decreased significantly over time, indicating that it can be excreted effectively. Except for the accumulation in the liver at the early stages, PDGFB-FMS shows higher accumulation in tumors than non-targeted FMS, demonstrating excellent tumor-targeting (Fig. 7e). Further research was also conducted into the pharmacokinetics of PDGFB-FMS, FMS, and Gd-DTPA. At p.i. 10 h, the residual concentrations of FMS and Gd-DTPA were only 18.9% and 4.6%, respectively, while PDGFB-FMS was 23.8%. Moreover, PDGFB-FMS had the longest half-life in the blood (t_1/2_ = 3.68 h) compared to FMS (t_1/2_ = 2.88 h) and Gd-DTPA (t_1/2_ = 0.97 h) (Fig. 7f–h). Notably, at p.i. 72 h, PDGFB-FMS could be completely eliminated from the body, thereby avoiding the potential risks to the body posed by long-term residual [42]. To summarize, the superior biocompatibility of PDGFB-FMS and its prompt in vivo clearance facilitate its use in tumor molecular MRI.

## Conclusion

In conclusion, TME-activated “off–on” T_1_–T_2_ dual-mode MRI nanoswitch (PDGFB-FMS) has been successfully designed and fabricated. This novel nanoswitch structure is composed of a superparamagnetic Fe_3_O_4_ core and a paramagnetic Mn-doped silica shell, in which the T_1_ and T_2_ signals are suppressed due to the phenomenon of distance-dependent magnetic resonance tuning. When exposed to GSH and a low pH value, the T_1_–T_2_ dual-mode MRI signals are activated with significant T_1_ and T_2_ contrast enhancement. PDGFB-FMS has been utilized for the precise diagnosis of heterotopic and orthotopic tumors due to its increased SNR and tumor-specificity. Biosafety assessments further confirm the low toxicity of PDGFB-FMS. In addition, due to its rapid biodegradability, PDGFB-FMS can be excreted out of the body in a timely manner, posing no potential risk to the body. This study, therefore, provides a promising strategy for the development of intelligent MRI contrast agents to achieve accurate diagnosis of tumors.

## Supplementary Information

**Additional file 1: Figure S1.** The hydrodynamic sizes of different samples. **Figure S2.** TG curves, XPS spectra, FT-IR spectra, and zeta potential of different samples. STEM image of FMS. **Figure S3.** Hydrodynamic size variation of PDGFB-FMS in different media. **Figure S4.** TEM images of FMS after different treatments and corresponding hydrodynamic size distribution. **Figure S5.** T_1_WI and T_2_WI of FMS. **Figure S6.** r_1_ and r_2_ values of FMS. **Figure S7.** r_1_ value of free Mn^2+^ ions at 3.0 T, hydrodynamic size distribution of FMS-1 to FMS-5, r_1_ linear fit curves and r_2_ linear fit curves of FMS-1 to FMS-5. **Figure S8.** N_2_ adsorption–desorption isotherms and corresponding pore size distribution of FS, and FMS-1 to FMS-5. **Figure S9.** T_1_–T_2_WI of FMS with different Fe_3_O_4_ to Mn^2+^ ratios. **Figure S10.** Fe content of 4T1 cells. **Figure S11.** CLSM observation: the internalization of PC-3 cells. **Figure S12.** Colocalization and particle uptake ratios of 4T1 cells. **Figure S13.** Representative color-coded T_1_ and T_2_ map images of 4T1 tumor-bearing mice and T_1_ and T_2_ map values analysis. **Figure S14.** Cytotoxicity of PDGFB-FMS against PC-3 cells. **Figure S15.** Blood routine examination of mice. **Figure S16.** The viabilities of THLE-3, 293 T, PC-12, and SH-SY5Y cells. **Table S1.** ∆r_1_ and ∆r_2_ value of FMS under different pH conditions. **Table S2.** ∆r_1_ and ∆r_2_ value of FMS under different GSH conditions. **Table S3.** In vitro ∆R_1_ and ∆R_2_ of 4T1 cells treated with PDGFB-FMS at the presence of different concentrations of GSH.
