# Supplementary material for: PDGFB-targeted functional MRI nanoswitch for activatable T1–T2 dual-modal ultra-sensitive diagnosis of cancer
Source: J Nanobiotechnology. 2023 Jan 6;21:9. doi: 10.1186/s12951-023-01769-7 (PMC9824934; doi:10.1186/s12951-023-01769-7)

**Additional file 1**

**PDGFB-targeted functional MRI nanoswitch for activatable T_1_-T_2_ dual-modal ultra-sensitive diagnosis of cancer**

Ya’nan Zhang^†,§^, Lu Liu^†,‡^, Wenling Li^†,‡^, Caiyun Zhang^†,‡^, Tianwei Song^†,‡^, Peng Wang^†,‡^, Daxi Sun^†,‡^, Xiaodan Huang^†,‡^, Xia Qin^†,‡^, Lang Ran^†,‡^, Geng Tian^†,*^, Junchao Qian^§,¶,*^, Guilong Zhang^†,‡,*^

^†^ School of Medical Imaging, Shandong Technology Innovation Center of Molecular Targeting and Intelligent Diagnosis and Treatment, Binzhou Medical University, Yantai 264003, P.R. China

^‡^ Institute of Biomedical Imaging Probe, Binzhou Medical University, Yantai 264003, P.R. China

^§^ Hefei Cancer Hospital, Anhui Province Key Laboratory of Medical Physics and Technology, Institute of Health and Medical Technology, Hefei Institutes of Physical Science, Chinese Academy of Sciences, Hefei 230031, P.R. China.

^¶^ Department of Radiation Oncology, School of Medicine, Shandong University, Shandong Cancer Hospital and Institute, Shandong First Medical University and Shandong Academy of Medical Sciences, Jinan 250117, Shandong, China

^*^ Correspondence: [tiangeng@bzmc.edu.cn](mailto:tiangeng@bzmc.edu.cn) (G.T.); qianjunchao@hmfl.ac.cn (J.Q.); [glzhang@bzmc.edu.cn](mailto:glzhang@bzmc,edu.cn) (G.Z.)

**Experimental Section**

**Materials.** Unless otherwise stated, all solvents and chemicals in this work were supplied from commercially available sources and used as received. Fe(acac)_3_ (98%), diethylene glycol (DEG, 99%), anhydrous sodium acetate (99.7%), tetraethylorthosilicate (TEOS, 99%), fluorescein isothiocyanate (FITC), glutathione (GSH), dimethyl sulfoxide (DMSO), N-hydroxysuccinimide (NHS), and N-ethyl-N′-(3-dimethylaminopropyl) carbodiimide (EDC) were purchased from Aladdin Biochemical Technology Co., Ltd. (Shanghai, China). mPEG-NH_2_ (MW=2000) and Sodium acrylate (95%) were purchased from Macklin Biotech, Co. (Shanghai, China). Ethylene glycol (EG, 99%) was achieved from Sinopharm Chemical Reagent Co., Ltd. (Shanghai, China). MnCl_2_· 4H_2_O was obtained from Zhiyuan Chemical Reagent Co. (Tianjin, China). Hoechst 33342 was purchased from Solarbio Science & Technology Co., Ltd. (Beijing). CCK-8 was obtained from Dojindo (Japan).

**Preparation of Fe_3_O_4_.** Fe(acac)_3_ (0.5 g, 0.001 mmol) was added to the mixture of EG (25 mL) and DEG (25 mL), and then stirred for 30 min at 80°C. Subsequently, sodium acrylate (1.5 g, 0.016 mol) was then added, followed by continuously stirring for 30 min. After that, sodium acetate (2 g, 0.02 mol) was added to the resulting solution, and the mixture was continuously stirred under stirring for another 45 min. Finally, the as-prepared solution was then sealed into a Teflon-lined stainless-steel autoclave and maintained at 200°C for 24 h. After the reaction finished, the black product was washed three times with alcohol and deionized water, collected by centrifugation, and then stored in distilled water for further use.

**Synthesis of FS.** Firstly, Fe_3_O_4_ solution (10 mL, 1.6 mg/mL) was dispersed in a mixed solution of distilled water (20 mL) and ethanol (140 mL, 2.45 mol) along with ultrasonication treatment. Thereafter, the concentrated ammonia (5 mL, 0.13 mol) was added to the above solution, and then the mixture was stirred at 250 rpm/min for 30 min. Afterward, the mixed solution of TEOS (3 mL, 1.56 mmol) and ethanol (8 mL, 0.14 mol) was dropped into the mixed solution and stirred continuously for 30 h. Finally, the nanoparticles were collected repetitively three times using a magnet, and washed with alcohol and deionized water.

**Fabrication of FMS.** Typically, FS (0.5 g) was uniformly dispersed in the solution (30 mL) containing MnCl_2_· 4H_2_O (30 mg, 0.15 mmol) and CH_3_COONa (20 mg, 0.24 mmol), and then the pH of the solution was adjusted to neutral. Subsequently, the mixed solution was transferred into Teflon-lined stainless-steel autoclaves and heated to 80°C, and the reaction was kept for 24 h. Then, the reacted process were repeated for 6 times in succession. Finally, the obtained solution was collected using a magnet and washed three times with alcohol and deionized water.

**Synthesis of PDGFB-FMS.** PDGFB cycle peptide (10 mg) was dissolved in 10 mL DMSO solution (0.13 mol), followed by adding EDC (50 mg, 0.32 mmol) and NHS (50 mg, 0.43 mmol). The mixed solution was stirred for 1.5 h at room temperature. Then, NH_2_-mPEG-NH_2_ polymer (10 mg, 5 μmol) was added to the above solution and continuously stirred for another 4 h. Next, FMS (10 mg) was added and continuously stirred overnight. Finally, the nanoparticles were centrifuged at 14000 rpm for 10 min and washed with the distilled water.

**pH and GSH-triggered Mn^2+^ release from PDGFB-FMS.** The PDGFB-FMS particles (5 mg) were dispersed in different pH solutions (10 mL, pH 7.4, 6.5, 5.5, 4.5) or different GSH concentrations of solution (10 mL, 5 mM, 10 mM, 20 mM), respectively. Then, the mixed solutions were shaken at 37°C, and the liquors were collected at different time intervals. Meanwhile, the liquors were centrifuged, and Mn content in the supernatant liquid was analyzed by ICP-OES.

**The r_1_ and r_2_ value measurements.** T_1_ and T_2_ relaxation time of PDGFB-FMS were examined by a series of inversion-prepared fast spin-echo sequence and a multiecho spin echo (MESE) sequence under 3.0 T scanner, respectively. Different concentrations of PDGFB-FMS were incubated with various GSH concentrations (0, 5, 10, and 20 mM) and different pH solutions (pH 7.4, 6.5, 5.5, and 4.5) before MRI. For T_1_ imaging, the parameters were as follows: TR/TE = 150, 300, 600, 1000, 2000, 4000, 8000/13 ms；FOV = 210 × 210 mm；Matrix size = 232 × 232；slice thickness = 3.0 mm (5 slices, gap=0). For T_2_ imaging, the parameters were as follows: TR/TE = 5000/10, 20, 30, 40, 50, 60, 70, 80, 90, 100 ms；FOV = 210 × 210 mm；Matrix size = 232 × 232；slice thickness = 3.0 mm (5 slices, gap=0).

**Cell viability assay.** To assess cytotoxicity of PDGFB-FMS, the viability of THLE-3 and 293T cells were examined via the CCK-8 assay. Briefly, THLE-3 and 293T cells were plated in a 96-well plate with a density of 2 × 10^4^ cells/well and then treated with different concentrations of PDGFB-FMS (0 to 140 µg/mL) for 24 h. Subsequently, the media was removed, and the cells were treated using 10% CCK-8 reagent for another 1 h. Finally, the absorption was detected using microplate reader at a wavelength of 450 nm.

**Cell uptake.** The internalization process of FMS and PDGFB-FMS were investigated using ICP-OES and CLSM. 4T1 cells were seeded in 60 mm culture plates with a density of 2 × 10^6^ cell/well. Subsequently, the cells were treated with different concentrations of FMS and PDGFB-FMS for 4 h. Meanwhile, the cells were also treated with FMS and PDGFB-FMS at a certain concentration of 10 μg/mL for 1, 2, and 4 h. Afterward, the media were removed and the cells were washed three times with cooled PBS and centrifuged for 5 min at 1000 rpm/min to collect cells into the tube. Finally, the concentrated nitric acid (1 mL) was used to digest the cells and then Mn^2+^ content in the solution were determined by ICP-OES.

For CLSM observation, FITC were rationally labeled into PDGFB-FMS and FMS, based on previously reported [1]. Then, 4T1 cells were seeded in the confocal glass plates at a density of 5 × 10^5^ cell/well, and incubated with FMS and PDGFB-FMS at the same method with ICP-OES investigation. Next, the cells were stained by Hoechst 33258 for 30 min at 37°C in the dark and rinsed with PBS for three times. Finally, the cells were imaged directly by a confocal microscope (Zeiss, LSM880).

***In vitro* MRI relaxation properties.** Cell MRI was performed using a 3.0 T MRI scanner. Briefly, the THLE-3 and 4T1 cells were cultured with FMS and PDGFB-FMS (40 μg/mL) for different time (1, 2, 4, 8, and 12 h). Then, the cells were washed three times with PBS, and collected, and fixed in the tube using 0.5 % agarose solution. Afterwards, T_1_WI and T_2_WI of samples were collected using a series of inversion-prepared fast spin-echo sequence and a multiecho spin echo (MESE) sequence, respectively. Meanwhile, the T_1_WI and T_2_WI signal intensity of samples were quantitatively measured using MRIcro software. The parameters were as follows: For T_1_WI, the parameters were as follows: TR/TE = 150, 300, 600, 1000, 2000, 4000, 8000/13 ms；FOV=180 × 180 mm；Matrix size=200 × 200；slice thickness=3.0 mm (5 slices, gap=0). For T_2_WI, TR/TE= 5000/10, 20, 30, 40, 50, 60, 70, 80, 90, 100 ms, FOV=180 × 180 mm；Matrix size=200 × 200；slice thickness=3.0 mm (5 slices, gap=0).

In order to investigate GSH responsive ability, the 4T1 cells were firstly pretreated with different concentrations of GSH from 2 to 10 mM, and then further co-incubated with PDGFB-FMS for 8 h. After that, the cells were washed with PBS, trypsinized, centrifuged at 1000 rpm/min for 4 min, and then fixed in 1 mL of 1% agarose gel. The cells were imaged by 3.0 T MRI scanner using the same sequence and parameters with the mentioned analysis.

***In vivo* MR imaging.** All *in vivo* MR studies were performed using a 7.0 T scanner. All the animal experiments in our study were in accordance with the Ethics Committee Guidelines of Binzhou Medical University. Cancer-bearing mice were anesthetized using a 1-2% isoflurane-oxygen mixture before MRI. Then, T_1_WI and T_2_WI of mice were acquired at the axial planes before and after the injection of different samples. For T_1_WI, the parameters were as follows: TR/TE = 1,500/7 ms, slice thickness = 1 mm, slice spacing = 0 mm, FOV = 40 × 40 mm^2^, matrix = 256 × 256. For T_2_WI, the parameters were as follows: TR/TE = 5,000/40 ms, slice thickness = 1 mm, slice spacing = 0 mm, FOV = 40 × 40 mm^2^, matrix = 256 × 256. For T_1_ map imaging, the parameters were as follows: TR/TE = 5,000/8 ms, slice thickness = 1 mm, slice spacing = 0 mm, FOV = 40 × 40 mm^2^, matrix = 256 × 256. For T_2_ map imaging, the parameters were as follows: TR/TE = 5,000/9 ms, slice thickness = 1 mm, slice spacing = 0 mm, FOV = 40 × 40 mm^2^, matrix = 256 × 256. ∆SNR of the tumor was calculated via the following equation:

∆SNR= SNR_post_-SNR_pre_ /SNR_pre_ × 100%

Where SNR_pre_ was signal to noise ratio at preinjection of imaging probe. SNR_post_ was signal to noise ratio at postinjection of imaging probe. In addition, T_1_ and T_2_ maps were reconstructed using Paravision software, meanwhile, the T_1_ and T_2_ relaxation times were collected.

**Biodistribution of PDGFB-MS.** Tumor-bearing mice were intravenously injected with FMS and PDGFB-FMS at the dosage of 8 mg/kg. At different time intervals, the mice were sacrificed, the vital organs were excised, weighed, and then nitrated into transparent solution using concentrated nitric acid. After that, the resulting solutions were diluted and filtrated using ultra-filter membrane with 220 nm pore. Finally, Mn content of solutions were analyzed via ICP-OES.

**Pharmacokinetics of PDGFB-MS.**Tumor-bearing mice were intravenously injected with Gd-DTPA, FMS, and PDGFB-FMS at the dosage of 8 mg/kg. Subsequently, the feces and urine produced by mice were collected at different time intervals. Then, the collected feces and urine were nitrated using the concentrated nitric acid, and the resulting solution were diluted and filtrated using ultra-filter membrane with 220 nm pore. Finally, Mn or Gd content of solutions were analyzed via ICP-OES.

**Biosafety evaluation of PDGFB-MS.** Tumor-bearing mice were intravenously injected with FMS and PDGFB-FMS at the dosage of 8 mg/kg. After 7 days, the mice were sacrificed, and the major organs including the heart, liver, spleen, lung, and kidney were excised, fixed, cut into slices, and then stained with hematoxylin and eosin (H&E). Finally, the damage of vital organs were analyzed by pathologist.

**Reference**

1. Pan C, Lin J, Zheng J, Liu C, Yuan B, Akakuru OU, et al. An intelligent T_1_-T_2_ switchable MRI contrast agent for the non-invasive identification of vulnerable atherosclerotic plaques. Nanoscale. 2021;13(13):6461−6474.


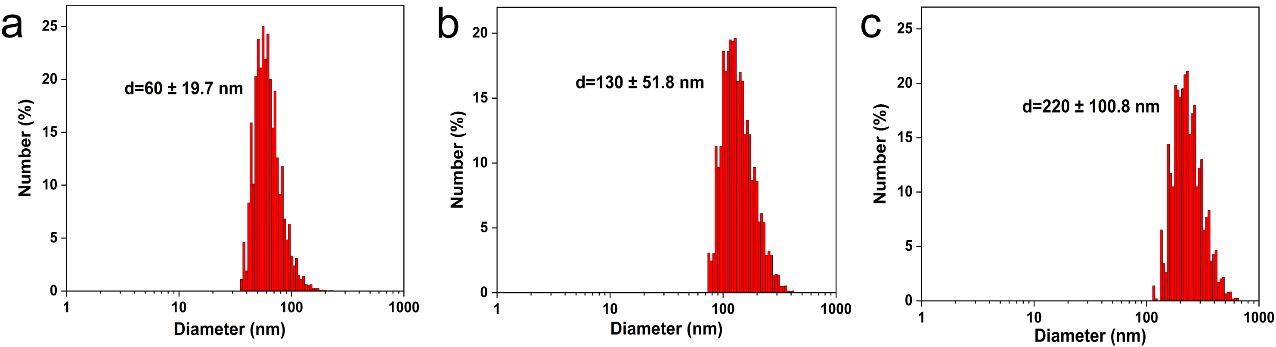


**Fig. S1** The hydrodynamic sizes of **a** Fe_3_O_4_, **b** FS, and **c** FMS.


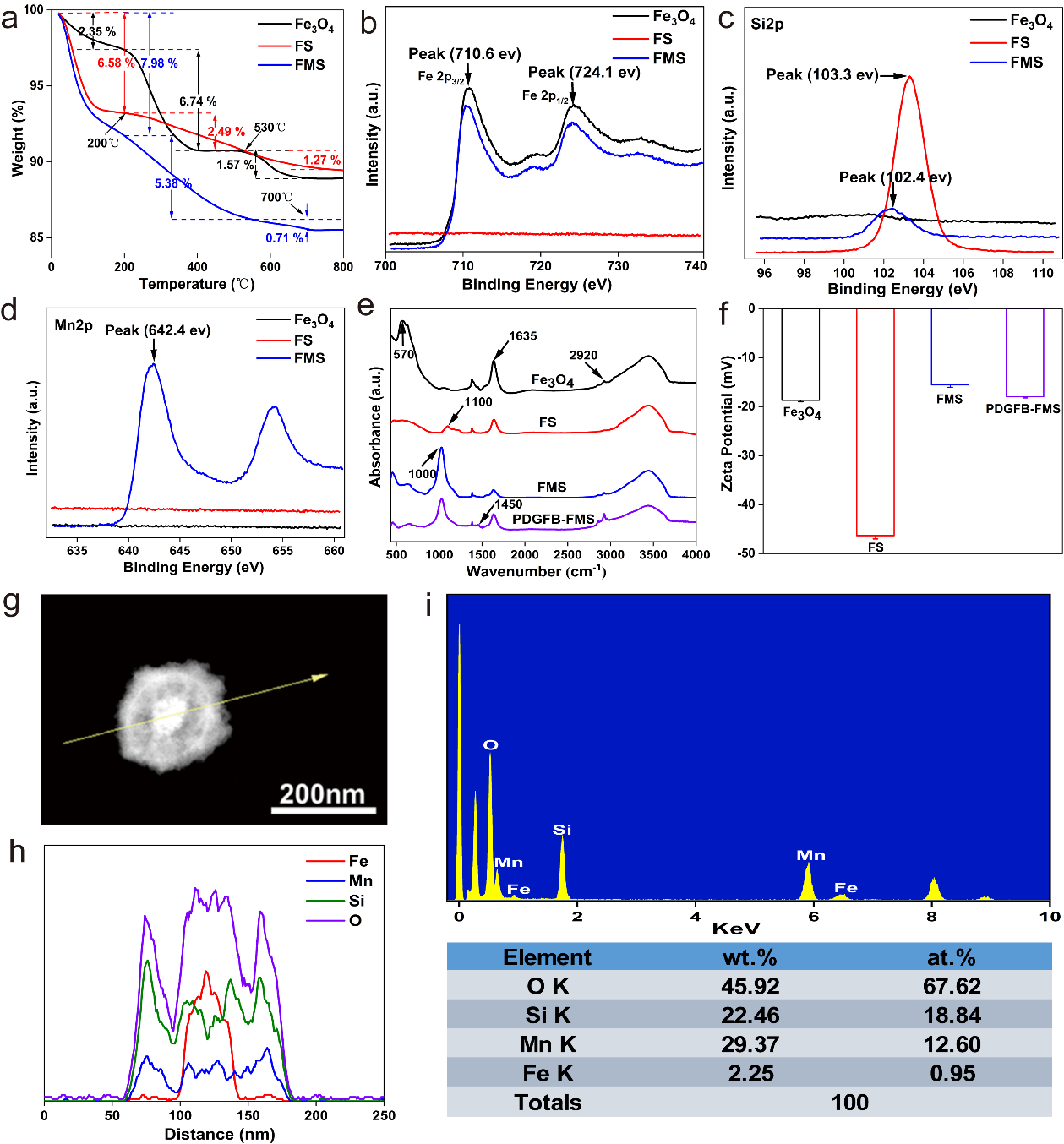


**Fig. S2** **a** TG curves, **b** the Fe2p XPS spectra, **c** the Si2p XPS spectra, and **d** the Mn2p XPS spectra of Fe_3_O_4_, FS, and FMS. **e** FT-IR spectra, and **f** zeta potential of Fe_3_O_4_, FS, FMS, and PDGFB-FMS. **g** STEM image of FMS. **h** STEM-EDS line profiles of FMS indicated in **g**. **i** EDS spectrum and the detailed elemental composition of FMS.


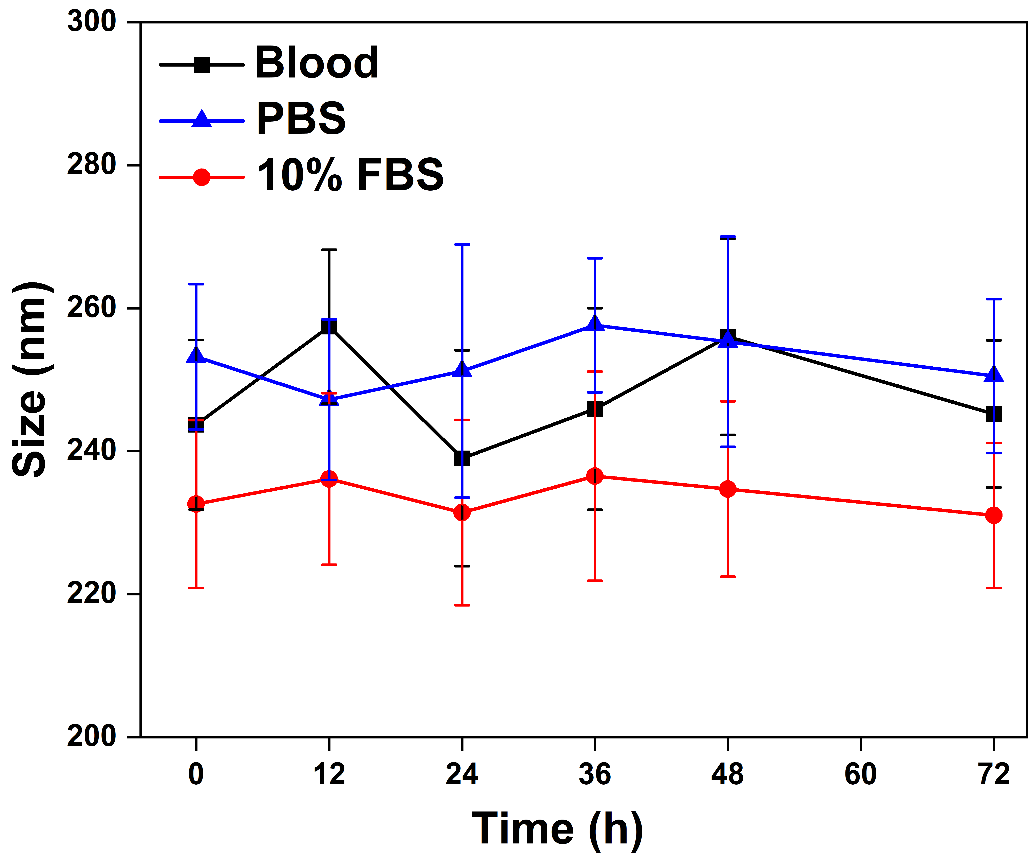


**Fig. S3** Hydrodynamic size variation of PDGFB-FMS in blood, PBS, and 10% fetal bovine serum (FBS).


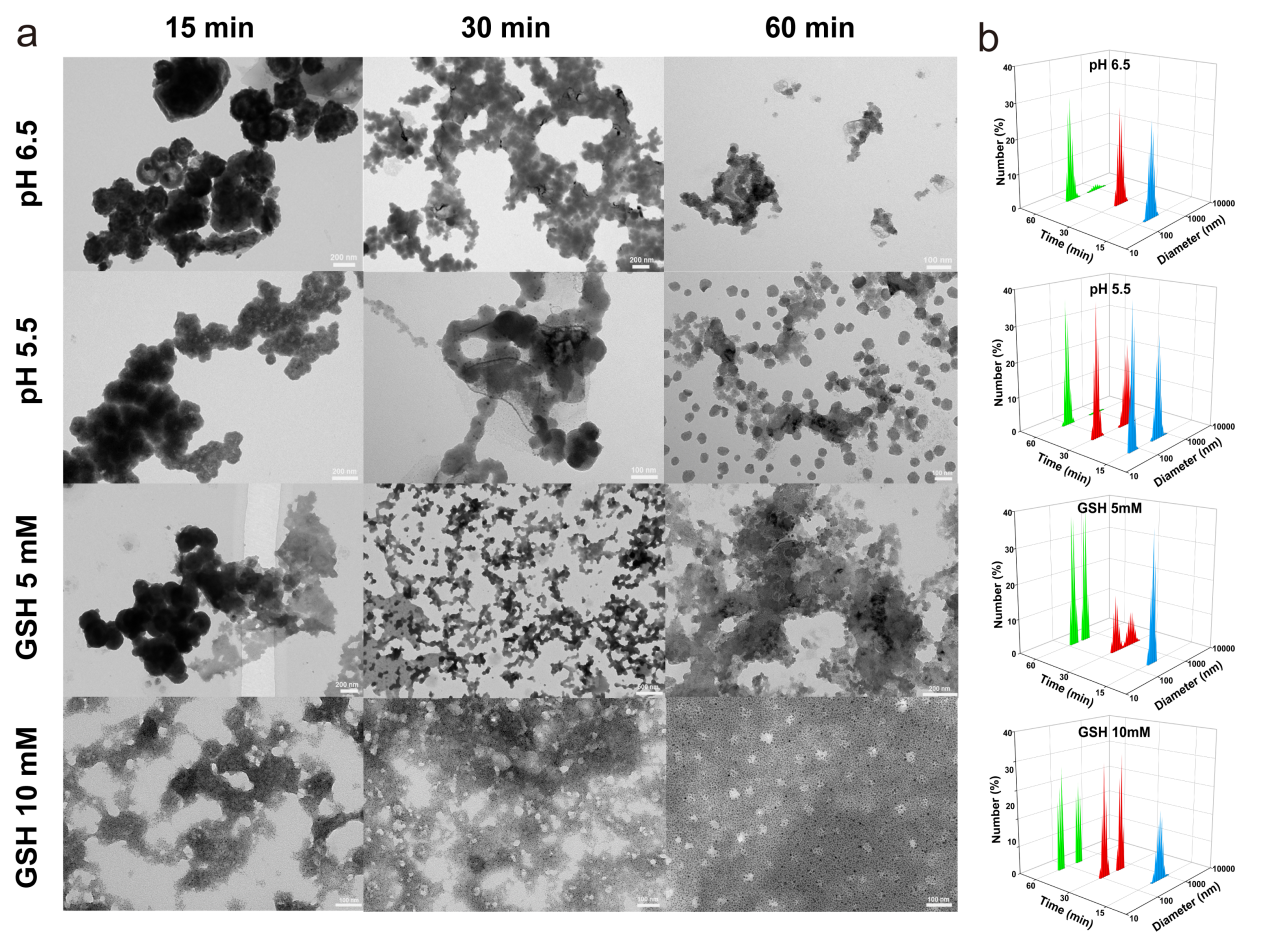


**Fig. S4** **a** TEM images of FMS after treatment with different pH and GSH conditions for 15, 30, and 60 min, **b** corresponding hydrodynamic size distribution.


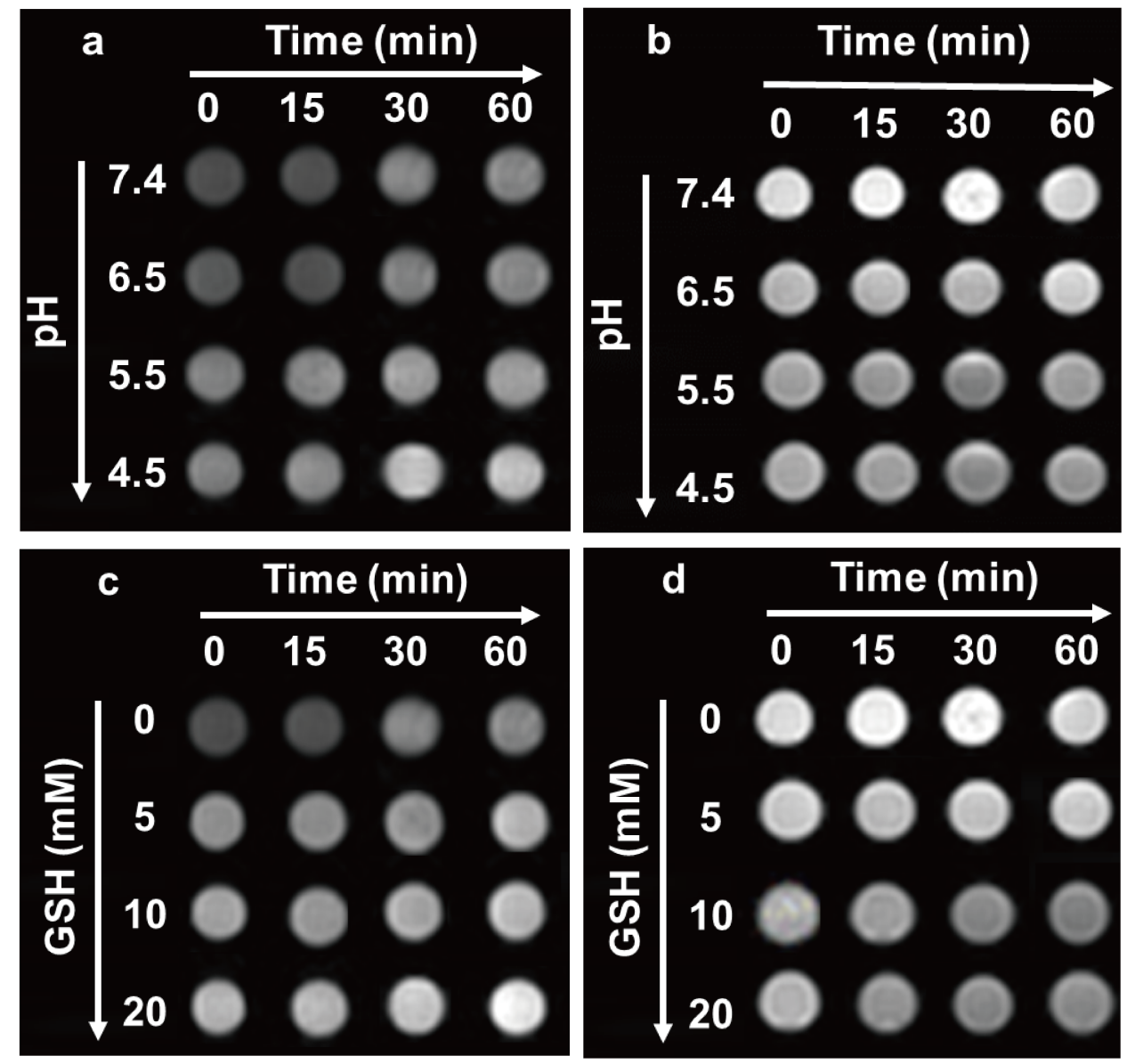


**Fig. S5** **a, c** T_1_WI and **b, d** T_2_WI of FMS as functions of pH value or GSH concentration and incubation time.


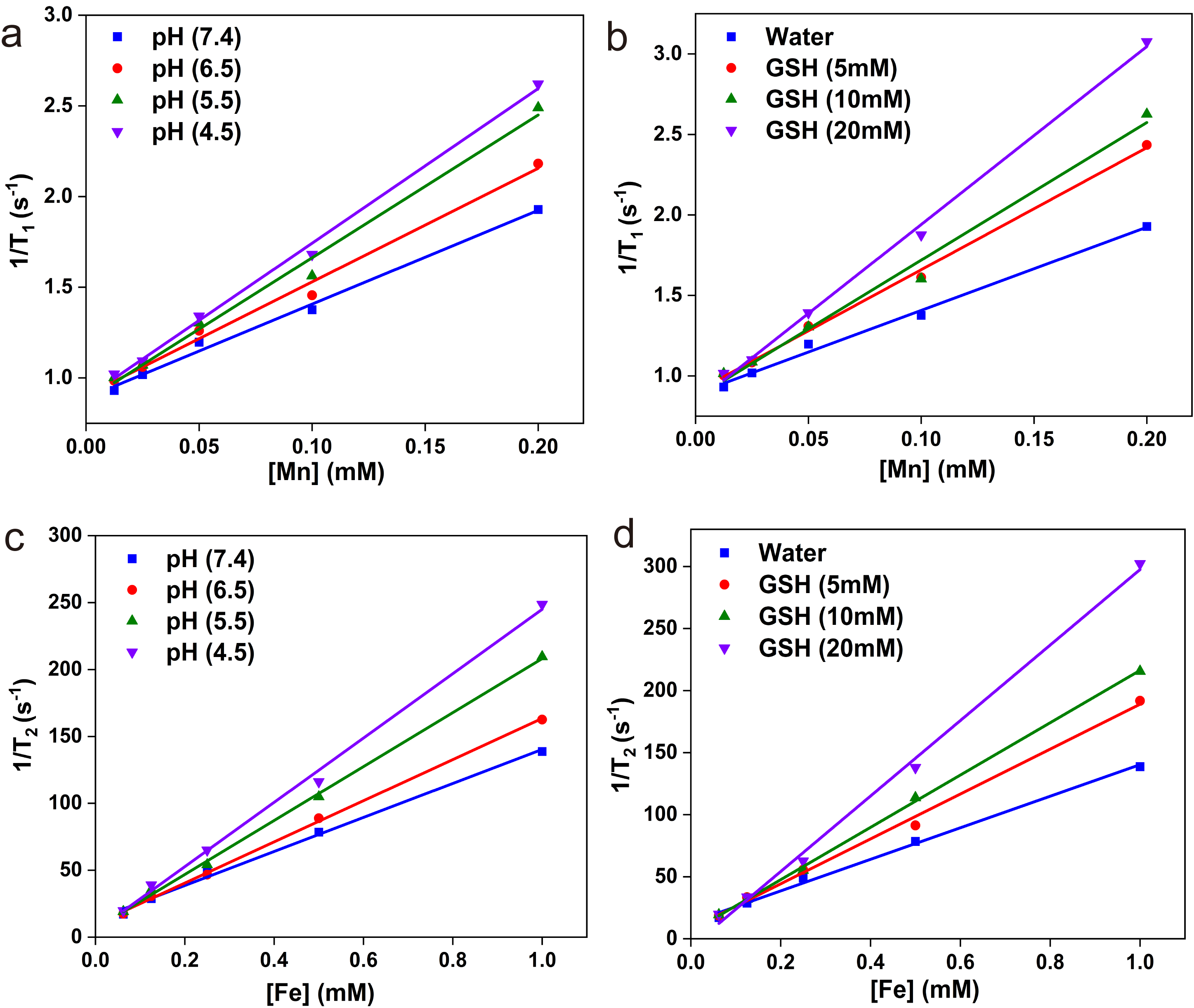


**Fig. S6** **a, b** r_1_ values and **c, d** r_2_ values of FMS under different pH (7.4, 6.5, 5.5, 4.5) and GSH concentrations (0, 5, 10 and 20 mM) solutions.


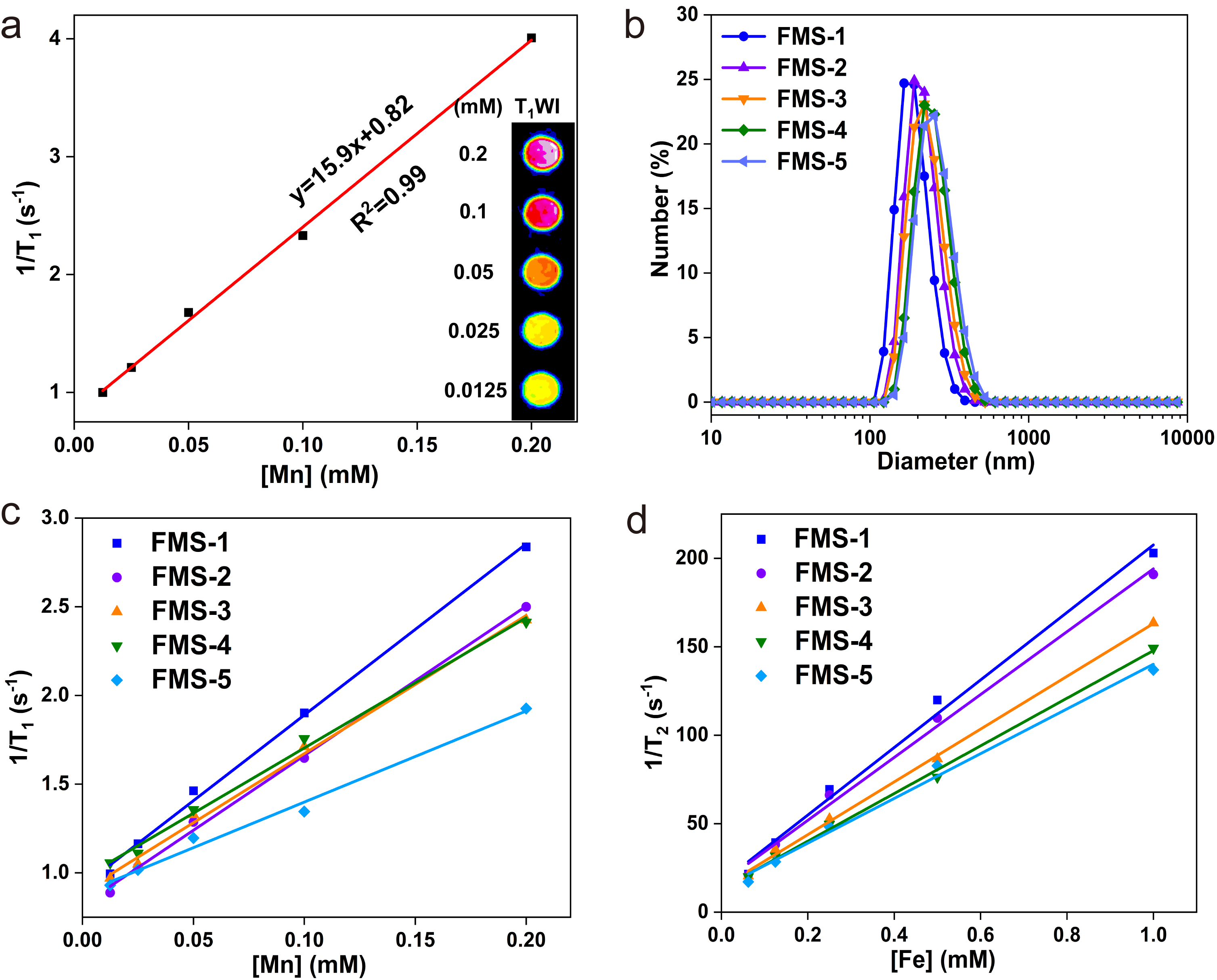


**Fig. S7** **a** r_1_ value of free Mn^2+^ ions at 3.0 T. **b** Hydrodynamic size distribution of FMS-1 to FMS-5. **c** r_1_ linear fit curves and **d** r_2_ linear fit curves of FMS-1 to FMS-5.


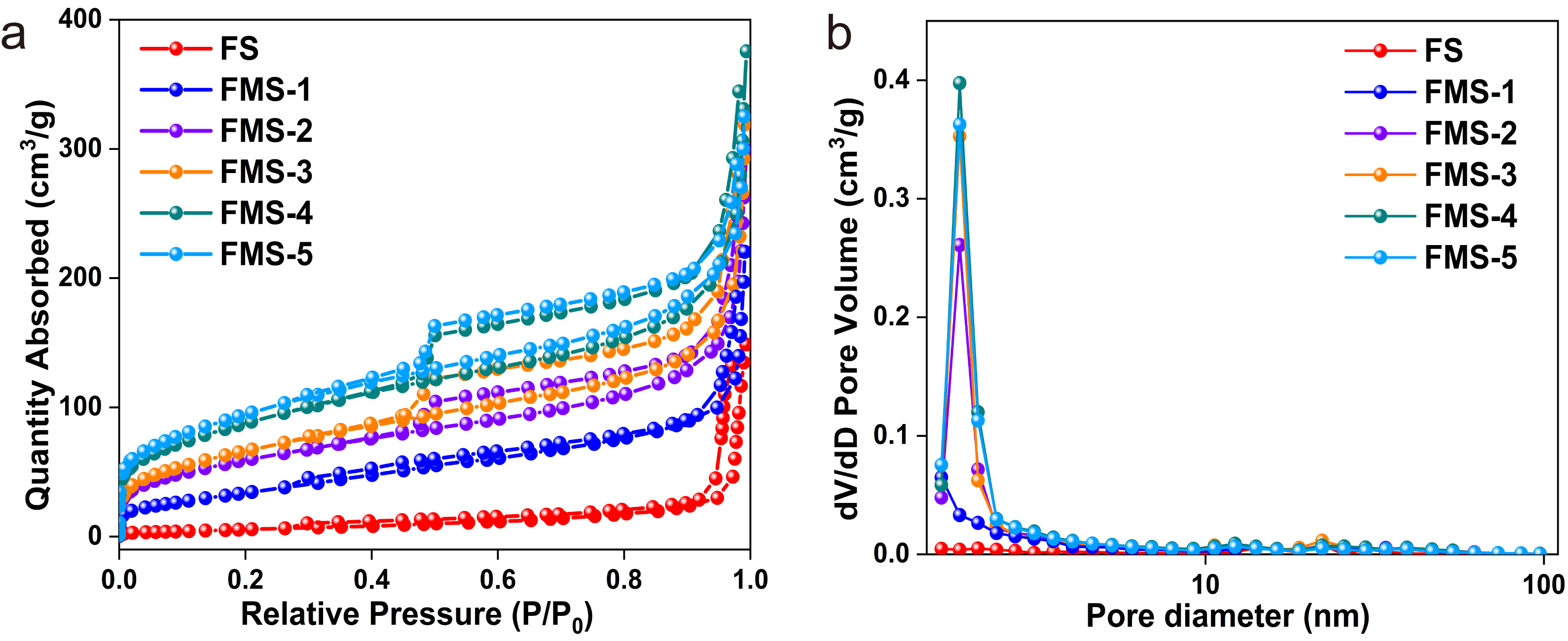


**Fig. S8** **a** N_2_ adsorption-desorption isotherms and **b** the corresponding pore size distribution of FS, and FMS-1 to FMS-5.


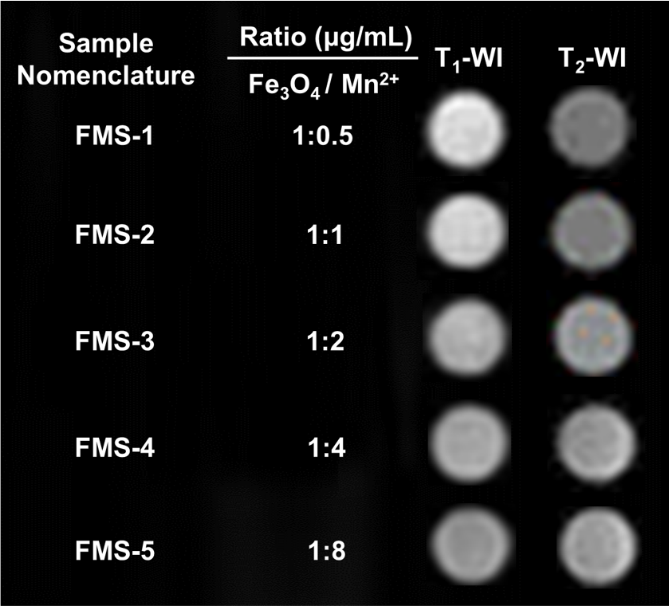


**Fig. S9** T_1_-T_2_WI of FMS with different Fe_3_O_4_ to Mn^2+^ ratios (1:0.5, 1:1, 1:2, 1:4, 1:8). The concentration of Fe_3_O_4_ was fixed at 1 μg/mL. The FMS nanoswitch showed obvious dual quenching in T_1_-T_2_ signals with the increase of Mn^2+^ doping ratio.


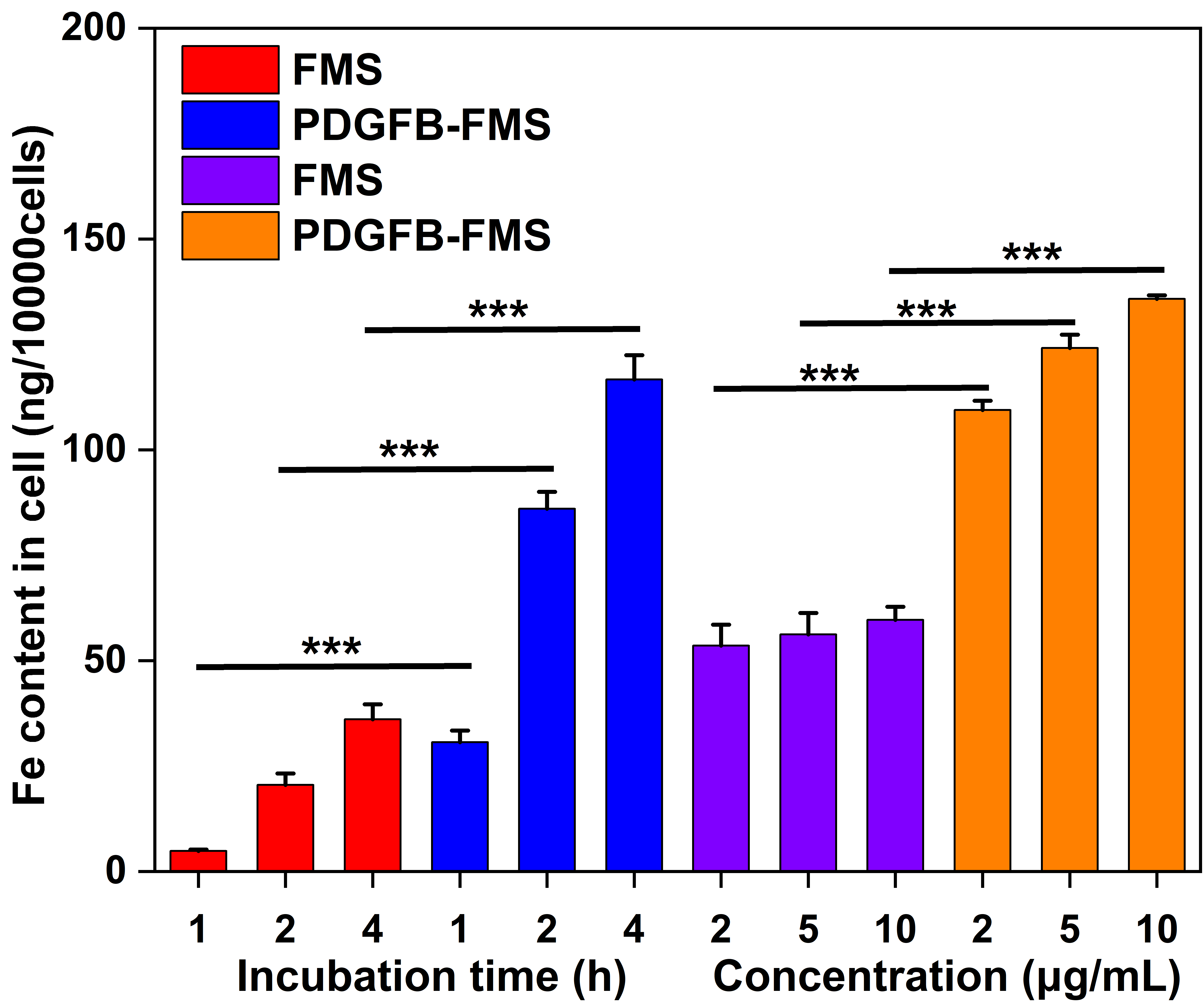


**Fig. S10** Fe content of 4T1 cells treated with FMS and PDGFB-FMS.


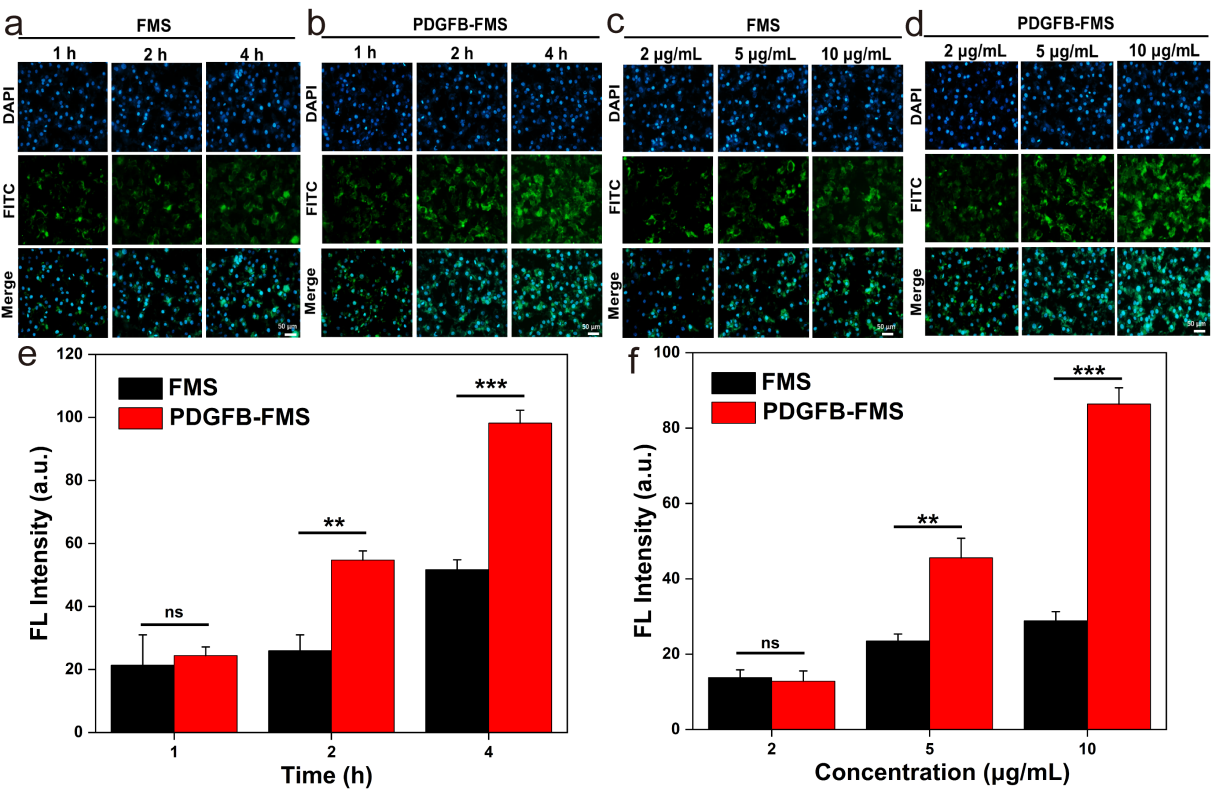


**Fig. S11 a, b** CLSM observation: the internalization process of PC-3 cells treated with FMS and PDGFB-FMS at a certain concentration of 10 μg/mL for 1, 2, and 4 h. **c, d** CLSM observation: the internalization process of PC-3 cells treated with different concentrations of FMS and PDGFB-FMS for 4 h. **e** Fluorescence intensity of PC-3 cells incubated with FMS (10 μg/mL) and PDGFB-FMS (10 μg/mL) at different time points. **f** Fluorescence intensity of PC-3 cells incubated with different concentrations of FMS and PDGFB-FMS for 4 h.


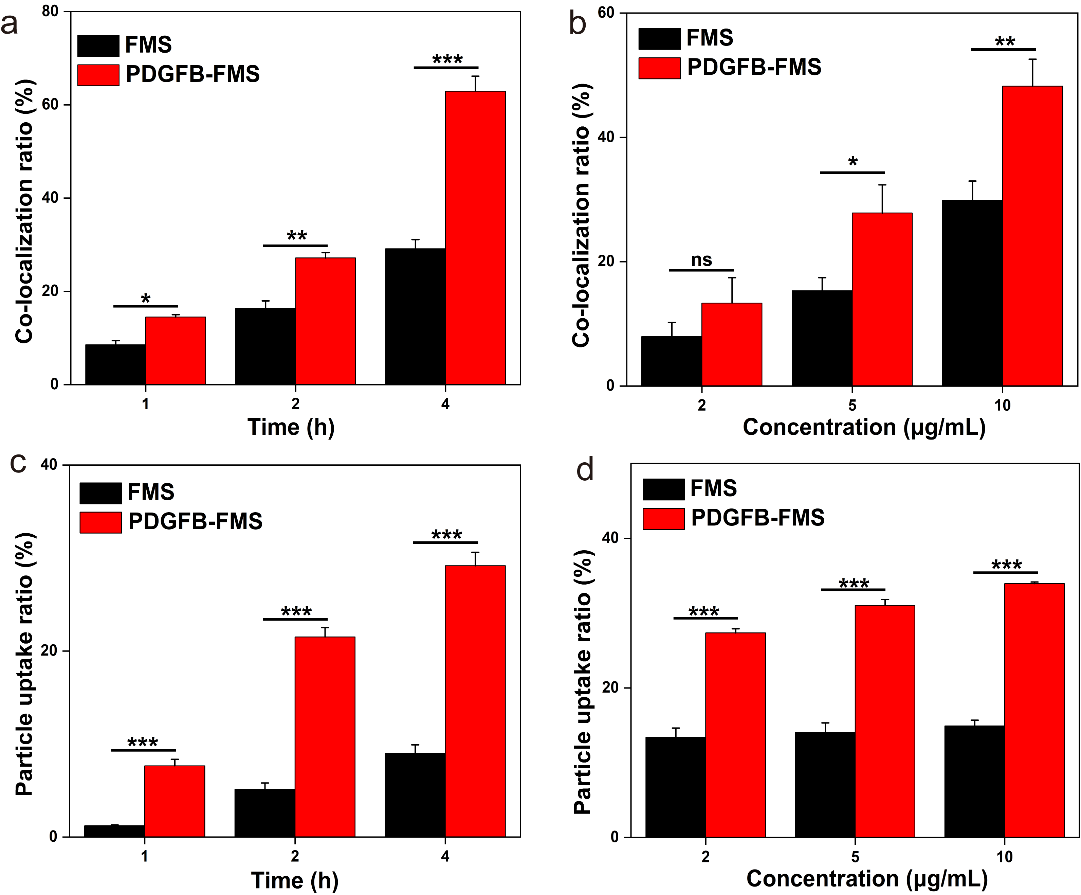


**Fig. S12 a** Colocalization and **c** particle uptake ratios of 4T1 cells incubated with FMS (10 μg/mL) and PDGFB-FMS (10 μg/mL) at different time points. **b** Colocalization and **d** particle uptake ratios of 4T1 cells incubated with different concentrations of FMS and PDGFB-FMS for 4 h.
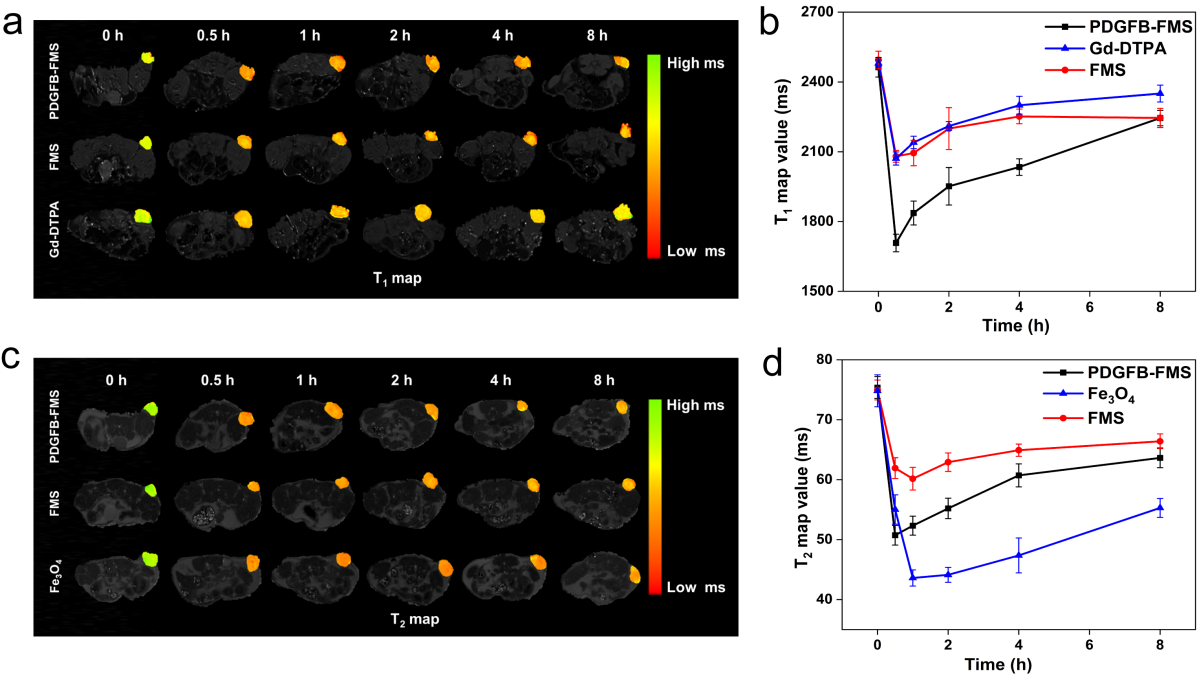


**Fig. S13** **a, c** Representative color-coded T_1_ and T_2_ map images of 4T1 tumor-bearing mice injected with different samples and **b, d** Average T_1_ and T_2_ map values analysis corresponding to **a, c**, respectively.


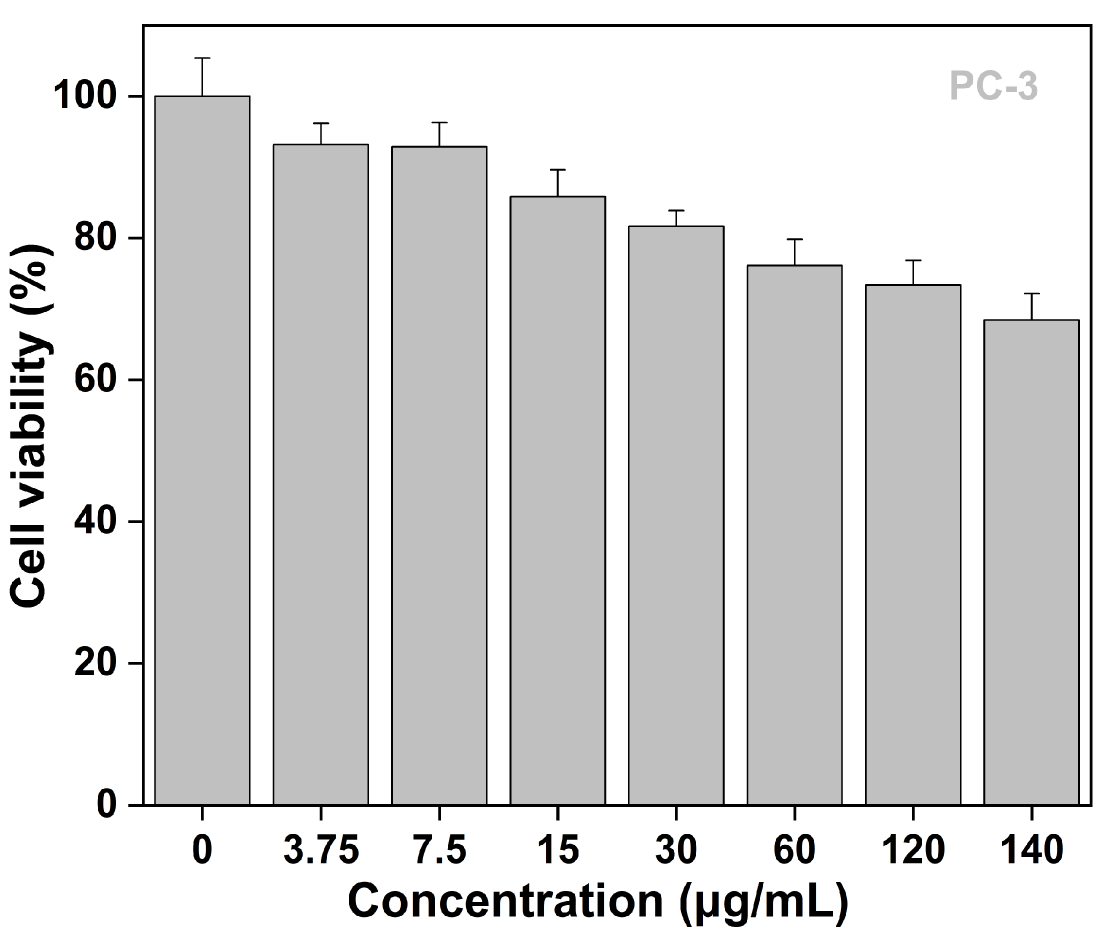


**Fig. S14** The cytotoxicity of PDGFB-FMS against PC-3 cells.


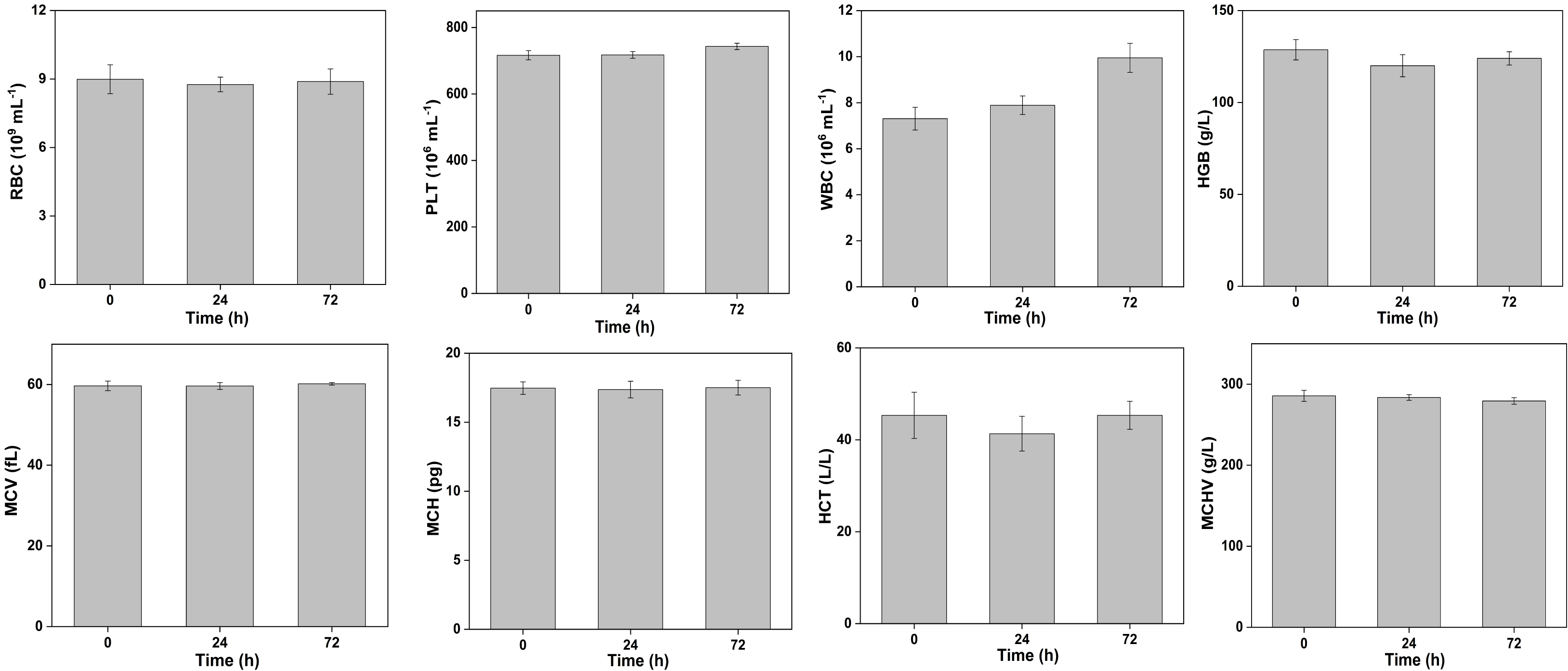


**Fig. S15** Blood routine examination of mice treated with PDGFB-FMS for 24 and 72 h.


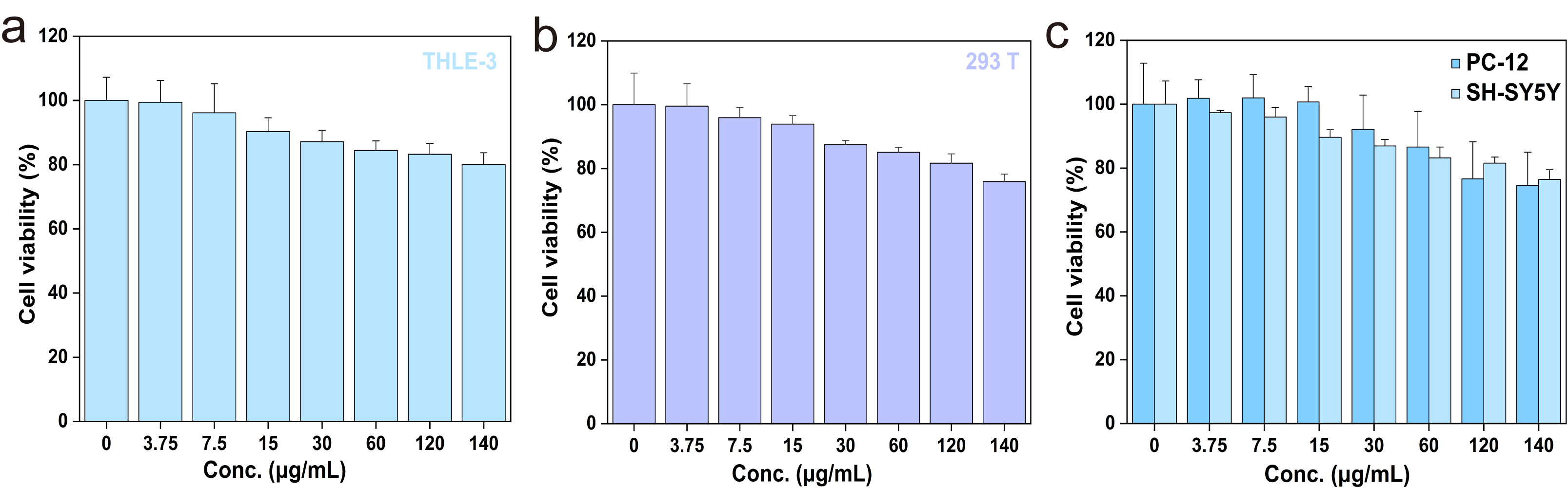


**Fig. S16** The viabilities of **a** THLE-3, **b** 293T, **c** PC-12, and SH-SY5Y cells treated with PDGFB-FMS for 24 h.

**Table S1.** ∆r_1_ and ∆r_2_ value of FMS under different pH conditions.


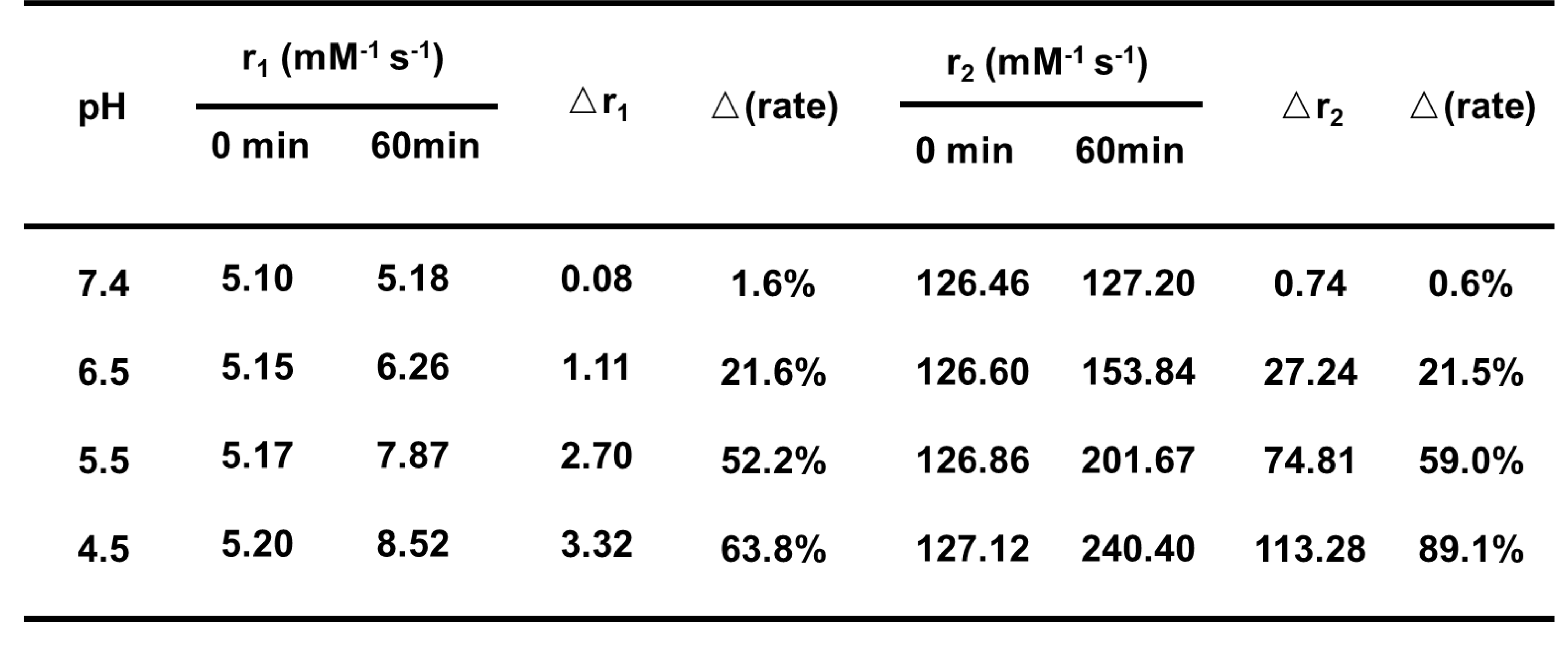


**Table S2.** ∆r_1_ and ∆r_2_ value of FMS under different GSH conditions.


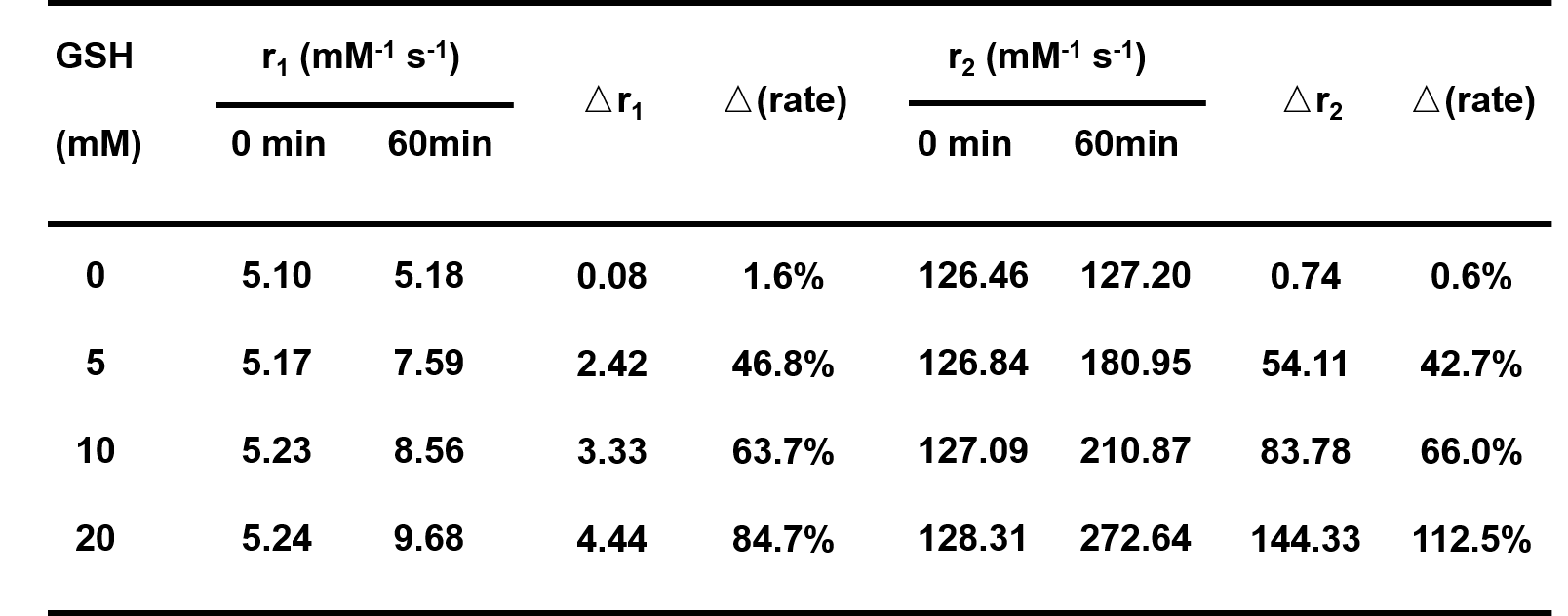


**Table S3.** In vitro ∆R_1_ and ∆R_2_ of 4T1 cells treated with PDGFB-FMS at the presence of different concentrations of GSH.


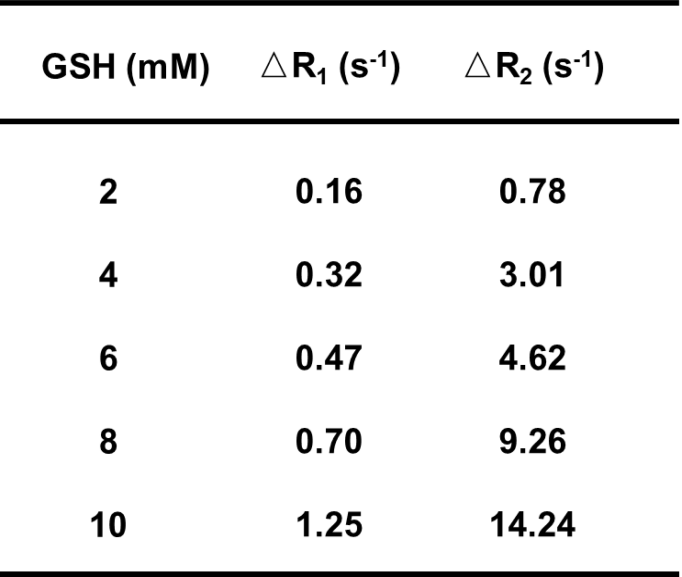

Supplement: Supplementary file 1 — Additional file 1: Figure S1. The hydrodynamic sizes of different samples. Figure S2. TG curves, XPS spectra, FT-IR spectra, and zeta potential of different samples. STEM image of FMS. Figure S3. Hydrodynamic size variation of PDGFB-FMS in different media. Figure S4. TEM images of FMS after different treatments and corresponding hydrodynamic size distribution. Figure S5. T1WI and T2WI of FMS. Figure S6. r1 and r2 values of FMS. Figure S7. r1 value of free Mn2+ ions at 3.0 T, hydrodynamic size distribution of FMS-1 to FMS-5, r1 linear fit curves and r2 linear fit curves of FMS-1 to FMS-5. Figure S8. N2 adsorption–desorption isotherms and corresponding pore size distribution of FS, and FMS-1 to FMS-5. Figure S9. T1–T2WI of FMS with different Fe3O4 to Mn2+ ratios. Figure S10. Fe content of 4T1 cells. Figure S11. CLSM observation: the internalization of PC-3 cells. Figure S12. Colocalization and particle uptake ratios of 4T1 cells. Figure S13. Representative color-coded T1 and T2 map images of 4T1 tumor-bearing mice and T1 and T2 map values analysis. Figure S14. Cytotoxicity of PDGFB-FMS against PC-3 cells. Figure S15. Blood routine examination of mice. Figure S16. The viabilities of THLE-3, 293 T, PC-12, and SH-SY5Y cells. Table S1. ∆r1 and ∆r2 value of FMS under different pH conditions. Table S2. ∆r1 and ∆r2 value of FMS under different GSH conditions. Table S3. In vitro ∆R1 and ∆R2 of 4T1 cells treated with PDGFB-FMS at the presence of different concentrations of GSH. [file 12951_2023_1769_MOESM1_ESM.docx]
